# Technical reliability of genotyping SNPs for forensic DNA phenotyping using SNaPshot- and MPS-based assays

**DOI:** 10.1007/s00414-025-03709-6

**Published:** 2026-01-08

**Authors:** Annica Gosch, Katja Anslinger, Jana Naue

**Affiliations:** 1 Institute of Legal Medicine, University Hospital of Cologne, Cologne, 50823 Germany; 2 Institute of Legal Medicine, LMU, Munich, 80336 Germany; 3 Institute of Forensic Medicine, Medical Center- University of Freiburg, Freiburg, 79104 Germany; 4 Faculty of Medicine, University of Freiburg, Freiburg, Germany

## Abstract

In recent years, several countries have undergone changes in their legal framework, now explicitly allowing the analysis of genetic markers for the purpose of forensic DNA phenotyping (FDP). Consequently, laboratory workflows for the analysis of appearance informative single nucleotide polymorphisms (SNPs) have been established in several laboratories. Currently, the HIrisPlex-S marker panel and webtool are the most widely used set of appearance informative SNP markers and statistical model used for phenotype predictions. However, many different laboratory protocols are employed for SNP genotyping, mostly using either massive parallel sequencing or single base extension technology. For the GEDNAP and TrACE proficiency tests, FDP modules were introduced in 2021 and 2023. These represented the first instances in which identical samples were analyzed by a fairly large number of laboratories, each of them employing their own laboratory-validated protocol. While mostly consistent phenotyping results were obtained, discrepant genotyping results were observed for some of the analyzed HIrisPlex-S-SNPs in BNC2, OCA2, and TYR. By performing a systematic in-silico analysis of commonly used primer sequences and sequencing the flanking regions of target SNPs in the affected samples from the collaborative exercises, we were able to identify primer binding site mutations, amplification of off-target products and overlap of SBE primers as risk factors for (analysis method-dependent) genotyping discrepancies. While the impact on phenotyping results was minor to negligible in all cases reported here, the issues uncovered by this in-depth analysis may provide a basis for improvements towards more consistent results in the future.

**Supplementary Information:**

The online version contains supplementary material available at 10.1007/s00414-025-03709-6.

## Introduction

An extensive body of research has shown that information about externally visible characteristics (EVCs), such as an individual’s eye, hair and skin colour, are encoded in single nucleotide polymorphisms (SNPs) [1, 2]. Predictions on EVCs can be obtained from a limited number of SNPs, which can be analysed from minute amounts of DNA such as recoverable from forensically relevant biological traces [3–5]. In cases where forensic STR profiling does not result in a match with a database or reference profile, obtaining information on EVCs of a trace donor, commonly referred to as Forensic DNA Phenotyping (FDP), may provide new investigative leads and help narrow down the group of potential suspects [1].

Following technical advances in the field of FDP, several countries have changed their legal framework, now explicitly allowing the prediction of certain EVCs from forensically relevant biological traces. In 2019, changes were made to the German Code of Criminal Procedure (ger.: “Strafprozessordnung”) now explicitly allowing for a prediction of a person’s eye colour, hair colour and skin colour as well as age from biological traces [6]. In Austria, FDP can legally be performed since 2018 [6] and most recently, Switzerland revised their law in 2023, now allowing for a prediction of eye colour, hair colour, skin colour, age and the biogeographic ancestry of a trace donor [7].

With the changes of the legal situation, several laboratories in these German-speaking countries implemented FDP workflows. Additionally, two German organisers of proficiency tests for forensic laboratories (German DNA Proficiency Test - GEDNAP [8, 9] and Trace Analysis Collaborative Exercise - TrACE [10, 11]) have started offering FDP modules, thus representing the first instances of collaborative exercises in which identical samples were analysed by a fairly large number of laboratories, each of them employing their own laboratory-validated analysis approach.

According to information provided by the proficiency test organisers as well as apparent from published research on FDP from different countries in the world (e.g.: [12–15]), most laboratories use the HIrisPlex-S marker panel and accompanying online prediction tool [16] as established by the Department of Genetic Identification of Erasmus MC [3–5, 17]. The marker panel consists of 41 appearance informative SNPs which can be detected either via the originally published SNaPshot™ assay (using the original PCR and SBE primers published in [5, 17] or modified versions thereof) or by using adapted assays in combination with massively parallel sequencing (MPS). Breslin et al. [18] modified the HIrisPlex-S assay for use on the two MPS platforms commonly used in forensic genetics, IonTorrent (HPS-MPS-ION) and MiSeq(FGx) (HPS-MPS-MiSeq panel). The primer sequences for use on the MiSeq(FGx) instrument were newly designed but mainly overlapping with the original SNaPshot™ assay (59 of 68 primer sequences are identical) with the addition of the necessary MPS-tag for subsequent index primer addition (Supplementary Table 1). In contrast, the primers for use with the IonTorrent platform were commercially designed by Thermo Fisher Scientific and therefore primer sequences were not provided in the publication [18]. It is thus not known whether and how these differ from the original SNaPshot™ PCR primer sequences. Additionally, PCR conditions of the original SNapShot™ assay were modified during method transfer to the MPS platforms.

The 41 HIrisplex-S-SNPs were also integrated by the European VISAGE consortium in two MPS-assays for use on the MiSeq(FGx) and the Ion Torrent platform targeting FDP as well as a set of ancestry-informative markers into the “VISAGE Basic Tool” [19–21]. The primers were custom designed for these assays, but sequences are not publicly available. The “IonAmpliSeq VISAGE Basic Tool Research Panel” can, however, be obtained from Thermo Fisher Scientific as an Ion AmpliSeq community panel [22]. Another panel with custom-designed primers available as an Ion AmpliSeq community panel is the “PhenoTrivium” panel, which comprises a total of 320 ancestry and appearance informative SNPs, including the 41 HIrisPlex-S-SNPs [23]. Nowadays, also ready-to-use commercial kits can be purchased. One of the first commercially available kits was the Verogen ForenSeq DNA SignaturePrep kit [24] that includes the eye and hair colour informative SNPs from the HIrisPlex-panel. The more recent specialized FDP assay, the Verogen ForenSeq Imagen Kit [25], targets all 41 HIrisPlex-S-SNPs. The Signature Prep Kit and Imagen Kit use the same primer pairs for the overlapping SNPs; which can be different to or modified from the original SNaPshot-HIris-Plex-S primer sequences (personal communication with Verogen/Qiagen).

In summary, all the available MPS solutions target the same set of 41 SNPs and the sequencing results can be checked in detail for quality and coverage. Primer sequences of commercial kits (including custom design assays) are generally not publicly available. It can however, be assumed and is known in some cases that modifications were (partially) introduced by the companies.

Phenotype predictions based on genotyping results of the 41 HIrisPlex-S appearance informative SNPs can be obtained from the HIrisPlex-S-webtool [16]. The webtool uses a model based on multinomial logistic regression, which generates prediction probabilities for three categories of eye colour (blue, intermediate, brown), four categories of hair colour (blond, brown, black, red) as well as two categories of hair shade (light, dark) and five categories of skin colour (very pale, pale, intermediate, dark, dark-black). According to the manual available from the webtool’s website [16], the eye colour model was trained on data from 9466 individuals (97,1% of whch are from European populations). The hair colour model was trained on data from 1878 individuals (82,6% of which are from European populations) and the skin colour model was trained on data from 1423 individuals taken from Walsh et al. 2017 (approximately 80% of which are from European populations) [26].

For the GEDNAP collaborative exercise, participating laboratories were instructed to use the prediction algorithm of their choice, whereas for the TrACE collaborative exercises, it was recommended and later required to use the HIrisPlex-S-webtool. Besides the webtool, a dedicated software for analysis of data generated with the VISAGE panel was developed [27, 28].

Taken together, currently a broad consensus exists regarding the minimal analysed marker set and statistical prediction model(s), but many different custom as well as commercial protocols for SNP genotyping are employed.

## Aim of study

The FDP collaborative exercises organised as part of the GEDNAP and TrACE proficiency tests represented the first instances in which identical samples were analysed by a fairly large number of laboratories, each of them employing their own laboratory-validated analysis approach. For the collaborative exercises in 2022 (GEDNAP) as well as 2023 and 2024 (TrACE), discrepant genotyping results for some of the analysed HIrisPlex-S-SNPs were observed (Table 1). The reasons for such discrepancies can be drop-outs in single laboratories due to low DNA quantity or quality or technical issues such as low coverage in an MPS run or low signal intensities in the analysis of the SNaPshot™ reaction by capillary electrophoresis. However, in all cases presented in Table 1, multiple laboratories were affected suggesting the presence of a systematic bias. We therefore aimed to investigate the reasons for these discrepancies. For this purpose, we performed a systematic analysis of genetic structures and variations potentially affecting binding of the HIrisPlex-S-PCR and -SBE primers by BLAST analysis and additionally assessed the presence of such variations in the affected samples from the collaborative exercises by sequence analysis of flanking regions. We subsequently discuss how genetic variations may affect the HIrisPlex-S SNP genotyping and implications for phenotype predictions.

**Table 1:** Detected discrepancies between laboratories for three collaborative exercises. The first genotype was later identified as correct

SNP (within gene)	Genomic location (GRCh 38)	Prof. test sample	Stated genotypes*	Stated by laboratories (count)
rs10756819 (BNC2)	chr9:16858086	GEDNAP 65 - individual B	GA, GG	8, 9
rs1470608 (OCA2)	chr15:28042975	TrACE 2023 - Ext. individual 2	GT, GG	9, 6
rs1126809 (TYR)	chr11: 89,284,793	GEDNAP 65 - individuals A, C	AA, AG	15, 4
		TrACE 2024 - Ext. individual 1	AA, AG	17,5

* (Partially) deduced from 0/1/2 –labelling of the HIrisPlex-S webtool

## Materials and methods

### Samples and phenotyping analyses for the collaborative exercises

Samples were sent to participating laboratories as part of the FDP and Extended Trace Analysis modules of the GEDNAP (2022) and TrACE (2023, 2024) collaborative exercises, respectively. For each of the collaborative exercises, samples from three different individuals were provided as dried blood spots on cellulose. DNA was extracted and phenotyping analysis was performed by each participating laboratory according to their own laboratory protocol. All participating laboratories submitted genotyping (as SNPs or 0/1/2-labelling according to the HIrisPlex-S webtool) and phenotyping (predicted eye, hair and skin colour) results and provided information on the employed laboratory procedure (SNaPshot™ analysis, various MPS assays) (Table 2).

**Table 2:** Reported genotypes, corresponding analysis technologies, and primer panels used. Information as available based on submission details provided by the laboratories

SNP	Prof. test sample	Stated genotypes/0/1/2-labelling	Technology used by reporting laboratories (count)	Primer panel used by reporting lab.
rs10756819 (BNC2)	GEDNAP 65 ind. B	**GA**	MPS (8)	Commercial panels
		GG	MPS (2) SNaPshot (7)	HPS-MPS-MiSeq panel (at least 1) [18] HIrisPlex-S*
		NA**	MPS and SNaPshot (1) SNaPshot (2)	
rs1470608 (OCA2)	TrACE 2023 ext. ind. 2	**GT**	MPS (9)	ForenSeq Imagen (V), VISAGE BT AmpliSeq Research (S5) (custom TFS), -AmpliSeq PhenoTrivium (custom TFS)
		GG	MPS (2) SNaPshot (4)	HPS-MPS-MiSeq panel [18] ForenSeq DNA Signature Prep Kit (V) HIrisPlex-S*
		NA	MPS	Position not analyzed
rs1126809 (TYR)	GEDNAP 65 ind. A and C	**AA**	MPS (15)	Commercial kits, HSP-MPS-MiSeq [18]
		AG	SNaPshot (4)	HIrisPlex-S*
		NA**	MPS and SNaPshot (1)	
	TrACE 2024 ext. ind. 1	**AA**	MPS (15) SNaPshot (2)	VISAGE BT panel (S5) HSP-MPS-MiSeq [18] ForenSeq Imagen (Verogen/QIAGEN) PhenoTrivium HIrisPlex-S*
		AG	SNaPshot (5)	HIrisPlex-S*
		NA**	MPS (1)	

**X**: correct genotype (as determined by subsequent analysis), *custom modifications to individual primer sequences possible as this information was not explicitly asked for, ** A reason for why the genotype was reported as NA was not provided (possible reasons: not covered in assay, not detected or inconclusive, typing error)

V: Verogen (now part of Qiagen); TFS: Thermo Fisher Scientific

### *In-silico* analysis of primer binding and amplification properties

To assess the impact of genetic structure and variation on primer binding and amplification properties, an in-depth in-silico analysis was performed for the original HIrisPlex-S primers (published in [5, 17]), which mainly overlap with the HPS-MPS-MiSeq panel [18]. When using the SNaPshot™ methodology, genotyping is commonly performed in two multiplex assays (labelled as assays 01 and 02 in Supplementary Tables 1 and 2), whereas all primers are usually united in a single multiplex assay for MPS analysis.

In a first step, we aimed to assess whether any known genetic variation, such as SNPs in the primer binding regions, might impact primer binding. For this purpose, the genomic regions targeted by each PCR primer pair were identified by the NCBI Primer-BLAST tool [29] (Database: Refseq representative genomes, Organism: Homo sapiens, accessed: 2025-09-01) and visualised in the NCBI Sequence Viewer [30]. The SBE primer binding region was identified by searching the SBE primer sequence within the genomic region flanked by the corresponding PCR primers. The presence of SNPs in the identified primer binding regions was then assessed by using the Variation Viewer: All dbSNP build 157 v2 SNPs were visualised in the “Live RefSNPs” track and allele frequencies were assessed by filtering the tabular variation data for SNPs with MAF information from the 1000 Genomes (1000G) project (Phase 3) [31, 32], which indicates the global minor allele frequency (MAF). All “common” variations with a 1000G MAF < 0.01 were noted in Supplementary Tables 1–3.

In a second step, we aimed to assess whether off-target binding of PCR and/or SBE primers could potentially lead to unwanted amplification and generation of off-target PCR/SBE products. For this purpose, all PCR primer pairs were submitted to the NCBI Primer-BLAST tool (Database: Refseq representative genomes, Organism: Homo sapiens) to identify potential off-target PCR amplification products. Off-target amplification products were considered “relevant” when they were < 1000 bp and had ≤ 3 mismatches per primer and considered “high-risk” when they were < 1000 bp and had a maximum of 1 mismatch per primer (Supplementary Tables 1–3). Both on-target and “high-risk” off-target amplification products were downloaded from the NCBI sequence viewer as FASTA files and the generation of off-target SBE products was assessed by performing a nucleotide BLAST [33] of all SBE primers against the downloaded FASTA files.

### PCR and sequencing for check of possible primer binding mutation

To investigate if polymorphisms within the primer binding regions of the original HIrisPlex-S system as well as likely primer binding regions of the commercial kits (no exact information available) are present in the collaborative exercise samples with discrepancies, primers directly surrounding the regions of interest (rs10756819 in BNC2, and rs1470608 in OCA2-amplicon 3, and rs1126809 in TYR) were designed (primer3plus). Marker information and primer sequences are provided in Table 3 . For comparison, the other two samples of each collaborative exercise were analysed as well.

**Table 3:** Targeted amplicon regions within the study and surrounding primers for characterization of the complete region. The same primers were used for PCR and sequencing

SNP (within gene)	Genomic location of targeted HIrisPlex-S-amplicon (GRCh 38)	Primer orientation	Primer sequence (5’−3’)
rs10756819 (BNC2)	chr9:16858064–16,858,139	Forward Reverse	TGCCAGGTAAACTGTAAAGCA ACCTCAAAAGGAAAGGGGGA
rs1126809 (TYR)	chr11:89284739–89,284,838	Forward Reverse 1 Reverse 2	TCAACATCTTTCCATGTCTCCAGA ACACTAGATTCAGCAATTCCTCTGA AGTCATAGCCCAGATCTTTGGA
rs1470608 (OCA2)	chr15:28042918–28,043,062	Forward Reverse	TTCACTTTCTCTCTCTGCCAACA GAATACCTCCCCAGGCTTTG

DNA was amplified in a 10 µL volume containing 1 µL 10xGold PCR buffer, 1 µL MgCl2, 0.4 µL GoldTaq polymerase (all TFS), 10 pmol of each primer, 0.9 µL dNTPs (200 µM of each dNTP (Bioline, Luckenwalde, Germany), 1 ng of DNA and water ad 10 µL.

The cycling protocol was 95 °C for 10 min, 15 cycles with 95 °C for 40 s, 64 °C for 1 min (−0.6 °C/cycle), 20 cycles with 95 °C for 40 s, 57 °C for 1 min, 68 °C for 40 s, final extension at 72 °C for 10 min. PCR reactions were purified by adding 2 µL ExoSap-IT (USB, Cleveland, OH, USA) followed by incubation at 37 °C for 30 min and 80 °C for 15 min.

The BigDye™ v1.1 Terminator kit (Thermo Fisher Scientific) was used for direct sequencing in final volumes of 5 µL consisting of 5xBD v1.1 Terminator Ready Reaction Mix, 5xBD Dye Terminator Sequencing Buffer, 0.2 µM sequencing primer (same as PCR primer), and 1 µL of PCR product. After purification of the sequencing reaction products (DyeEx96 column plate, Qiagen), capillary electrophoresis (POP6, 36 cm capillary) was run on a 3130*xl* (Applied Biosystems) with Foundation Data Collection Software v3.0, respectively. Sequencher 5.46 software (GeneCodes, Ann Arbor, MI, USA) was used to compare the results to the reference sequences of each genomic region (GRCh38.p14 Primary Assembly).

### Phenotype predictions

To assess the impact of incorrect genotyping results on phenotype predictions, phenotypes of all individuals for which discrepant results were obtained during the GEDNAP and TrACE collaborative exercises were predicted using the Hirisplex-Webtool [16] for both the correct and the incorrect genotypes for the respective SNPs.

## Results and discussion

### *In-silico* analysis of primer binding and amplification properties – general observations

A detailed summary of the In-silico analysis results is provided in Supplementary Tables 1–3 as well as supplementary files 1 and 2.

No common variants with a MAF > 0.01 (1000G global MAF) were found in the PCR primer binding regions. However, it needs to be taken into consideration that this filter in the NCBI variation viewer refers to the global allele frequency. Thus, among the many SNPs with a global MAF < 0.01 identified in the primer binding regions (Supplementary File 2), some may be (more) common in certain populations (cf. Table 4).

**Table 4:** Detected SNPs in primer binding regions

HIrisPlex-SNP	SNP in primer binding region	Global MAF*	Region with high MAF*
**rs10756819**	rs112356725 G > A	0.0042 (0.012)	European: 0.017 (0.014)
**rs1470608**	rs574307918 G > A	− (0.00036)	African American: - (0.0018)

*global MAF obtained from dbSNP [38] build 157 1000 Genomes (total: 5008 subjects), - no frequency information in respective database, in brackets () ALFA (aggregate allele frequency from dbGaP, release version 20250407153717, total: 40236 subjects (for rs112356725) and 14050 (for rs574307918))

For the SNaPshot™-based analysis method, it should be noted that the amplicons generated by the assay 01 primer sets 2 and 3 contain 3 and 6 SNPs respectively. There are thus a few SBE primers overlapping each other, for which other HIrisPlex-S-SNPs can be found within the primer binding regions (Supplementary Tables 2, 3), and which may thus impact each other’s genotyping results.

To address this issue, ‘wobble’ bases [34] could be introduced during SBE primer design, ensuring that a proportion of the SBE primers could efficiently bind to the target region independent of the SNP allele carried by the individual. However, no participating laboratory using the SNaPshot™ method describe having their SBE in this way.

The majority of PCR primers do not pose a high risk of generating additional off-target PCR products (Supplementary Table 1). There are, however, three primer pairs located within the two genes HERC2 and TYR, for which off-target PCR product generation was considered “high-risk”:

The assay 02 PCR primer sets 6 and 8 (amplifying regions around rs6497292 and rs1667394) are intended to amplify the genomic locations chr15: 28,250,982–28,251,131 and chr15: 28,285,002–28,285,131, respectively, within the *HECT And RLD Domain Containing E3 Ubiquitin Protein Ligase 2* (*HERC2*) gene. With a single mismatch for one primer of set 6 and one mismatch in each primer of set 8, the primer sets 6 and 8 potentially amplify six and three different regions on chromosome 15, respectively. In each case, this could lead to the generation of an off-target PCR product of identical length and high sequence similarity compared to the intended PCR product (Supplementary File 1). The high sequence similarity is explained by the fact that several partially duplicated paralogs of the HERC2 gene can be found on chromosome 15 [35, 36]. Stringent PCR conditions are thus important to avoid/reduce the off-target amplification. If nevertheless co-amplified, it would be possible to differentiate between the target and off-target PCR products in MPS-based genotyping as the PCR product sequences are not entirely identical. Within the HPS-MPS-MiSeq panel, the reverse PCR primer for the rs1667394 target was modified and now contains two mismatches to the off-target regions, thus reducing the risk of co-amplification.

For the SNaPshot™ assay, the probability of generating an off-target SBE product may also be considered low, as the rs6497292-SBE-primer can only bind to the respective off-target PCR products with at least two mismatches in the primer-binding region and the rs1667394-SBE-primer can only bind to the respective off-target PCR products with at least three mismatches, thus off-target SBE products will be generated (much) less efficiently than the intended SBE products.

**Fig. 1:**
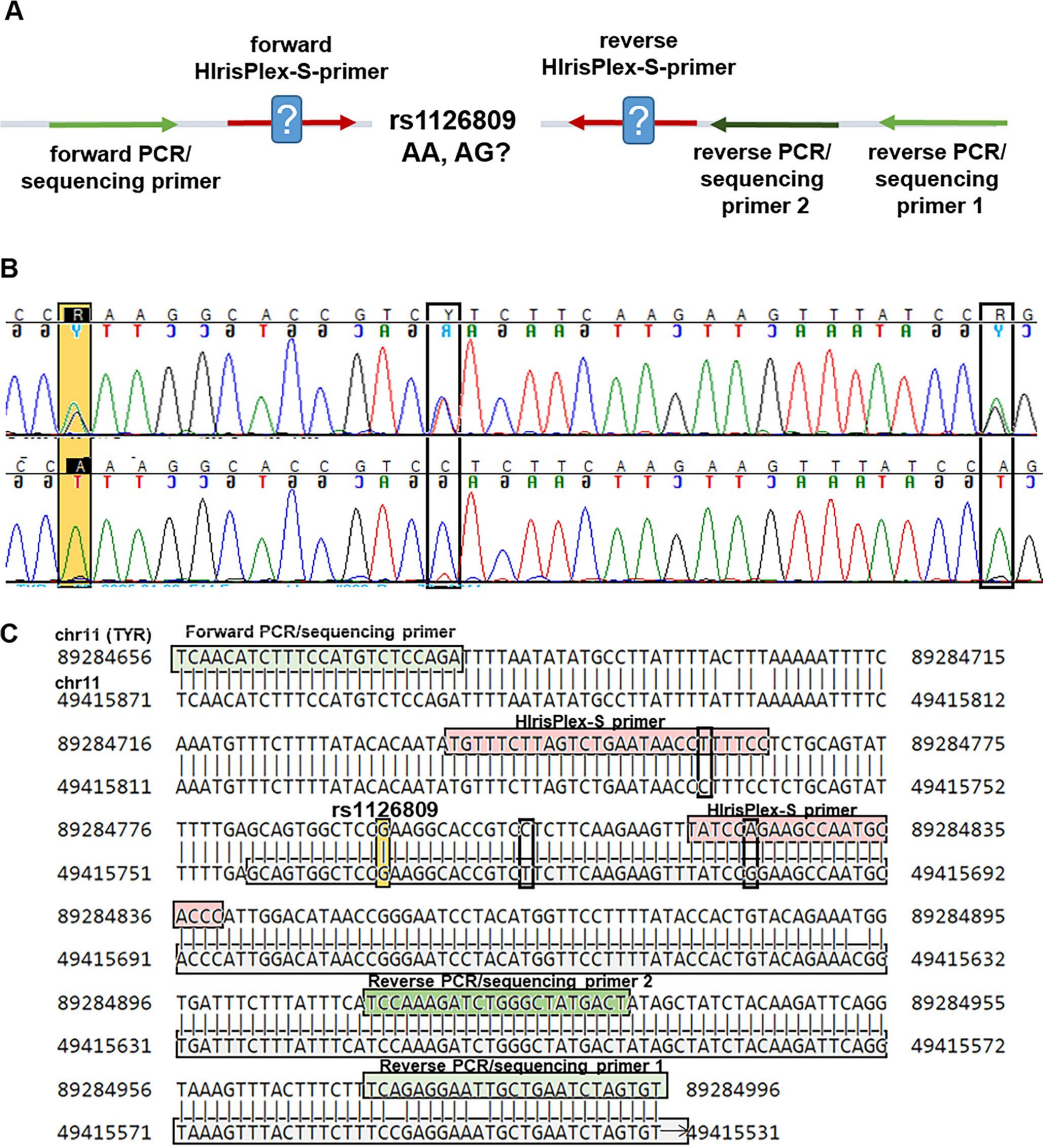
Determination of cause for wrong additional G detection in rs1126809 (TYR) (**A**) Schematic overview of experimental approach (**B**) Sequence of region of interest for TrACE 2024 extended individual 1 with primer pair F/R2individual B (upper pane) and F/R1 lower pane). Orange: SNP, black squares: additional discriminant positions between TYR and TYRL. (**C**) Sequence comparison of target (TYR) and off-target region containing TYRL (highlighted in grey). Orange/Black squares: positions as in chromatogram in B

The assay 02 PCR primer set 9 (amplifying a region around rs1126809) is intended to amplify the genomic location chr11: 89,284,739–89,284,838, lying within the *tyrosinase* gene (TYR). With a single mismatch for each primer, these primers potentially amplify a second region on chromosome 11 (49415689–49415788). This would lead to the generation of an off-target PCR product of identical length and high sequence similarity compared to the intended PCR product (Fig. 1 and Suppl. File 1). The high sequence similarity is explained by the fact that the off-target genomic region lies within the *tyrosinase like pseudogene* (TYRL) [37]. As the PCR product sequences are not entirely identical, it should be possible to differentiate between the target and off-target PCR products by MPS-based genotyping methods if not only the SNP of interest is considered during interpretation. However, as the rs1126809-SBE-primer can bind to the off-target PCR product without a single mismatch, the genotyping results obtained from SNaPshot™-based methods may be impacted by the off-target binding of the PCR primers.

Stringent PCR conditions can minimize the risk of co-amplification of off-target regions in these cases. Nevertheless, care must be taken as spurious amplification might be possible. Normally, extreme imbalances in allele coverage (MPS) or peak height (SNaPshot™) could be an indication for unintended off-target amplification. However, the interpretation may become more difficult in cases of low-quality/low-quantity samples which may exhibit increased allelic imbalances.

### Discrepant results for samples in FDP collaborative exercises

Discrepant genotyping results were observed for individual SNPs in the FDP modules of the GEDNAP (2022) and TrACE (2023, 2024) collaborative exercises. As these discrepancies were not only reported by a single or a few labs, but by a larger number of participating laboratories, a systematic investigation was performed.

Discrepant genotyping results obtained during the three collaborative exercises as well as a summary of the analysis methods employed by the respective participating laboratories are provided in Table 2.

For the GEDNAP 65 collaborative exercise, less detailed information was available, as participating laboratories were not yet required to provide raw data (electropherogram or MPS retrieved SNP information) at that time. Due to the observed discrepancies, this was changed for subsequent collaborative exercises (especially the TrACE exercise, first conducted in 2023).

Discrepant results for the SNP rs1126809 were obtained for three different individuals from two independent collaborative exercises. In-silico analysis had shown that the original HIrisPlex-S PCR primers amplifying the region around this locus can potentially amplify a second PCR product with one mismatch in each primer pair (Fig. 1, Suppl. File 1, Suppl. Tables 1, 2). This off-target PCR product contains a G nucleotide at the sequence position corresponding to rs1126809 within the intended PCR product. This G nucleotide will only lead to wrong genotyping results for individuals carrying a homozygous A allele at the true rs1126809 genomic location. It is thus plausible to assume that the incorrect reporting of the AG genotype by some laboratories was caused by unintended amplification of a genomic region within the TYRL pseudogene. Indeed, we were able to confirm this assumption by targeted sequencing analysis with newly designed primers (Table 3; Fig. 1): When the forward primer was used in combination with reverse 2, a second off-target PCR product was co-amplified, distinguishable from intended PCR product by sequence analysis. However, when the forward primer was used in combination with reverse 1, which carried two mismatches to the off-target-region, only the intended PCR product was generated, allowing for a verification of the AA as the correct genotype at rs1126809 (Fig. 1) for the GEDNAP 65 A and C individuals as well as TrACE 2024 ext. ind. 1.

**Fig. 2:**
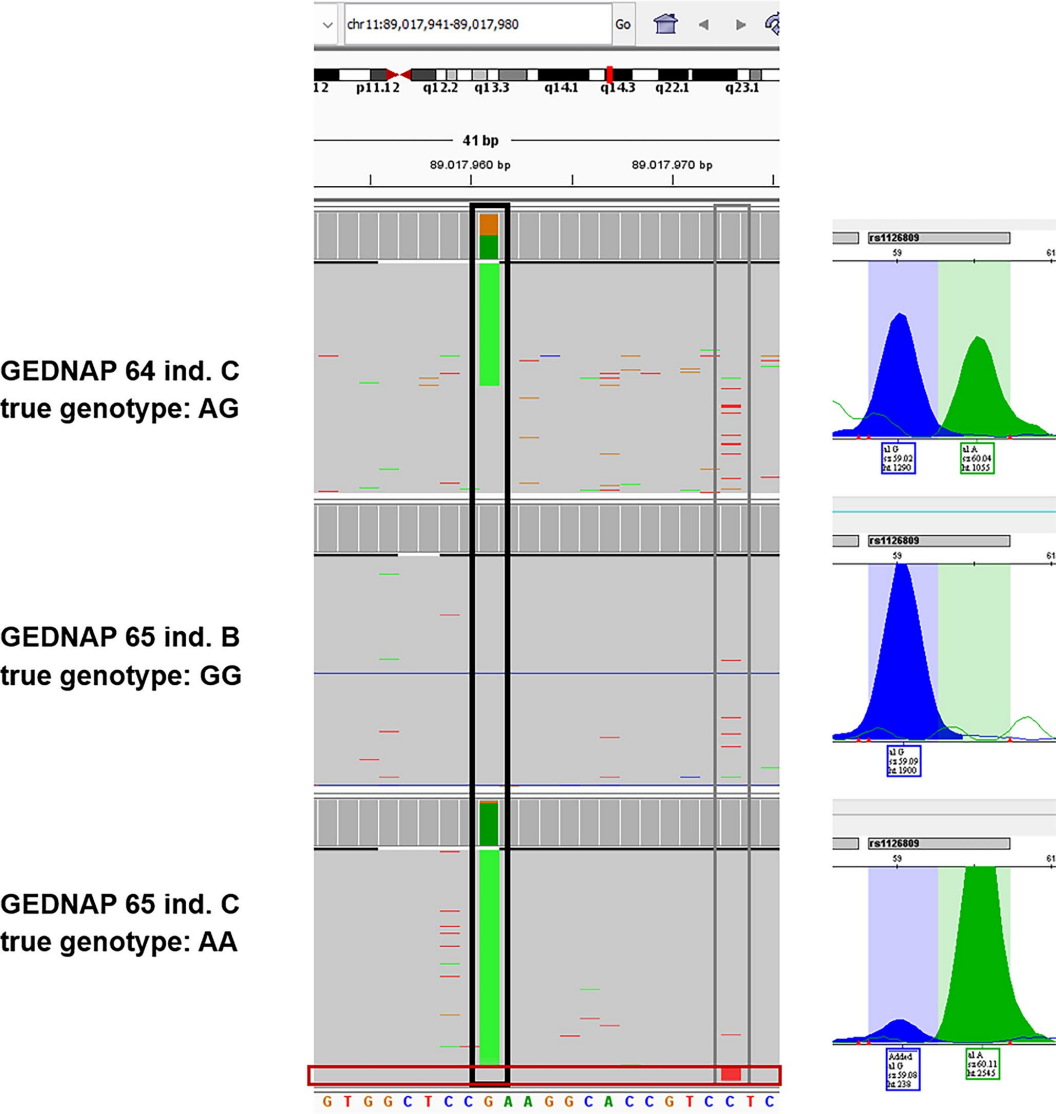
MPS (left) and SNaPshot (right) genotyping results for rs1126809 (TYR) For sample GEDNAP 65 individual C, the co-amplified G nucleotide can be connected in MPS to the off-target pseudogene TYRL which also has a T nucleotide at position chr11:89.017.973 (in contrast to the C in TYR) (highlighted in red box). In the samples GEDNAP 64 individual C and GEDNAP 65 individual B, some reads of the off-target TYRL can also be seen in the MPS data (as can by differentiated based on the T at chr11:89.017.973), which does however not impact genotyping results at position chr11:89.017.960 as the genotype of TYR also contains a G for these samples. The SNaPshot™ electropherogram also shows the additional G nucleotide of TYRL in GEDNAP65 individual C (It may be assumed that a proportion of the G signal for GEDNAP 64 individual C and GEDNAP 65 individual B also originates from the co-amplified TYRL, however, this cannot be differentiated in the SNaPshot™ data)

All MPS-performing laboratories obtained the correct results for rs1126809 (TYR) during the GEDNAP65 as well as TrACE 2024 (Table 2). It is not clear, if background co-amplification nevertheless occurred but remained below the reporting threshold, if the primers (off the commercial) kits are modified avoiding any co-amplification, or if the PCR conditions were strict enough to avoid any remaining annealing to the off-target region. Deeper analysis was possible for our own MPS analysis, which was based on the HPS-MPS-MiSeq panel, therefore using the original HIrisPlex-S primer. In all three affected samples (GEDNAP 65 individual A and C, and Trace 2024 ext. individual 1, the presence of the G nucleotide was noticeable. However, using the HPS-MPS analysis pipeline from Breslin et al. [18], only the AA genotype was called as final genotype. This is due to the strong allele imbalance of 88–95% A vs. 12 − 5% G in all three cases (e.g. final coverage after HPS-MPS analysis pipeline application for GEDNAP 65 individual C in duplicate analysis: A: 3483/1449, G: 211/46). Furthermore, MPS allowed a deeper investigation of the amplicon sequence itself, which led to the detection of multiple differences in the amplicon sequence compared to the TYR reference (cf. Figures 1 and 2), supporting the final decision to not report the G allele. Exemplarily, the IGV is presented for the ext. individual 1 of the TrACE 2024 exercise (Fig. 2).

Laboratories using the SNaPshot™-based genotyping methods obtained more divergent results in both collaborative exercises (Table 2). Again, the use of different amplification conditions might have led to reduced co-amplification of the off-target PCR product. Details regarding the PCR chemistry and employed amplification conditions were not obtained by the organisers of the collaborative exercises. Additionally, laboratories might have used different strategies (analytical thresholds, thresholds for heterozygote imbalance), which might have led to discrepant assessment of the co-amplified G-nucleotide. Indeed, in our own SNaPshot™ analysis (including the original HIrisPlex primer for the analysis of rs1126809), small G-nucleotide-peaks next to larger A-nucleotide peaks (71–91% A vs. 9–29% G in all three cases, e.g. for GEDNAP 65 individual C peak heights: A = 2545 rfu, G = 238 rfu), which led us to report the genotype as AA (Fig. 2).

**Fig. 3:**
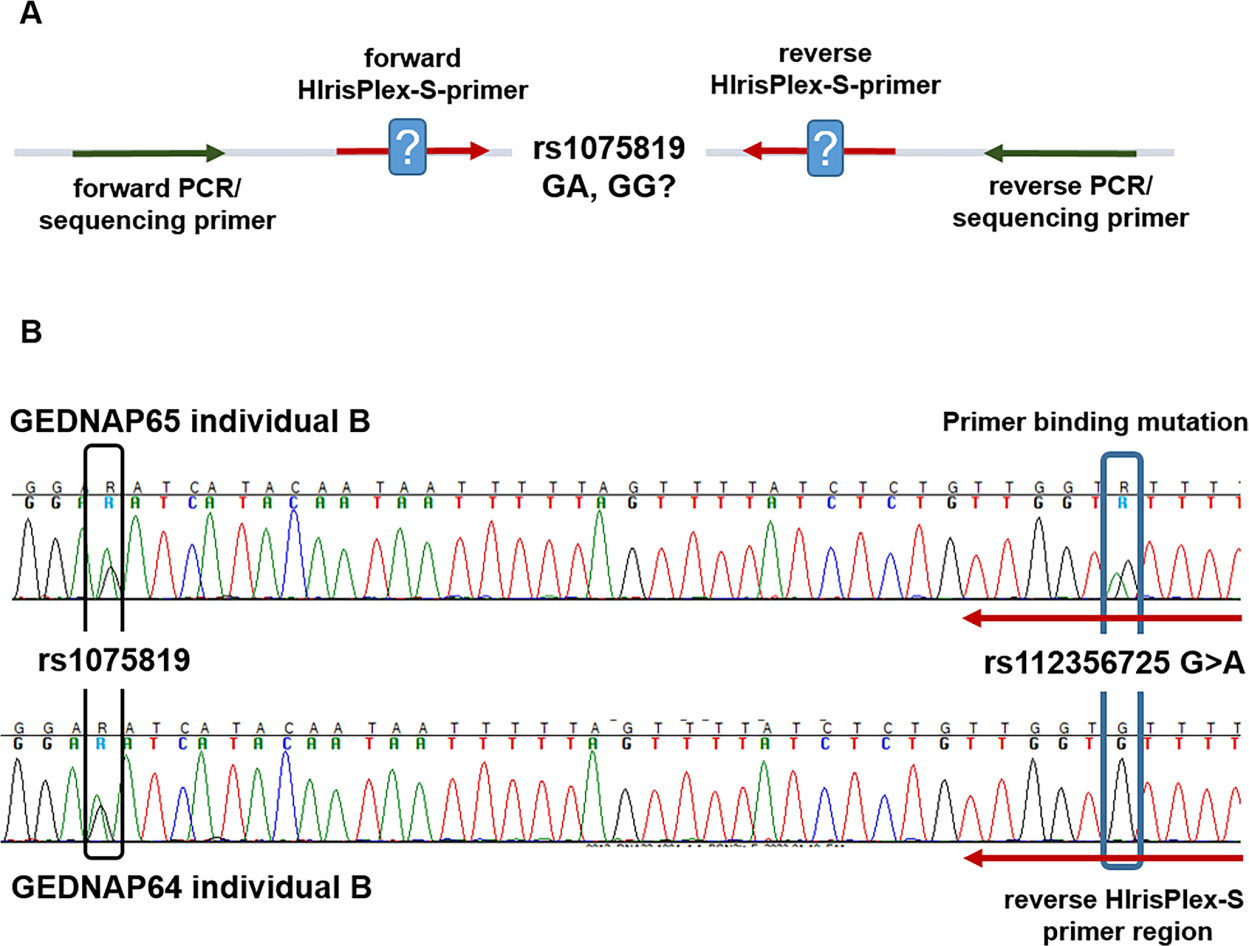
Determination of a primer binding mutation in the HIrisPlex-S primer region for rs10756819 detection (BNC2) (**A**) Schematic overview of experimental approach (**B**) Sequence of region of interest for GEDNAP65 individual B (upper chromatogram). In addition, the sequence of GEDNAP64 individual B (with the same AG genotype in the BNC2 SNP rs1075819) without a primer binding mutation is shown (lower chromatogram)

Discrepant results for the SNPs rs10756819 and rs1470608 were obtained for a single individual in one collaborative exercise, each. In-silico analysis did not suggest relevant off-target primer binding sites. No common variants (MAF ≥ 0.01) were discovered in the respective PCR or SBE primer binding locations. However, Sanger sequencing of the regions of interest revealed heterozygous primer binding site mutations within the HIrisPlex-S PCR primers for both rs10756819 (within the gene *basonuclin-2* (BNC2), (Fig. 3)) and rs1470608 (within the gene *oculocutaneous albinism* type 2 (OCA2)) (Table 4).

It was observed that all participants using the SNaPshot™-based genotyping method reported the incorrect homozygous genotyping results for rs10756819 and rs1470608, respectively. Although this information was not provided in detail, it can be assumed that all laboratories used either the original HIrisPlex-S primer panel or a custom assay based on these primers.

In case of MPS, nearly all laboratories obtained concordant results for all three samples. Exceptions are two laboratories which were not able to detect the heterozygote genotype for rs10756819 and rs1470608. One of these two was our own laboratory. Here, the HPS-MPS-MiSeq was used, which contains the same primers at the SNPs affected primer binding positions resulting in non-detection of the heterozygous alleles. Even after an in-depth sequence analysis, no background amplification of the second allele was detectable in our data. It is not known on which MPS-assay the second discordant result in BNC2 is based. The second discordant result in OCA2 was obtained in case of the use of the ForenSeq DNA Signature Prep Kit (Verogen). However, correct results were observed for the ForenSeq Imagen kit containing the same primers (personal communication from Verogen). This might be due to different calling settings or differences in the PCR conditions between laboratories.

Common SNPs in PCR primer binding regions should be avoided during primer design. However, avoiding the occurrence even of any rare SNP in a primer binding region is hardly possible. The possibility of such occurrences is more difficult to assess for assays for which the primer sequences are not publicly available.

### Impact of discrepant genotyping results on phenotype predictions

Lastly, we aimed to assess the impact of incorrect genotyping results on the obtained phenotype predictions. The three SNPs for which discrepant genotyping results were obtained during the collaborative exercises were all part of the skin colour prediction model only, thus eye and hair colour predictions were not affected. The visual phenotypes and the predicted probabilities obtained with the HIrisPlex-S-model [16] for each of the five skin colour categories depending on the genotype input are displayed in Table 5. In most cases, the incorrect genotyping result had a minor impact on phenotyping results as it did not lead to a different verbalisation of the predicted phenotype as suggested by the HIrisPlex-S-manual provided with the prediction webtool [16]. In two cases, a slightly larger difference was observed: For individual B from GEDNAP 65, a “dark to dark-black” skin colour would be predicted with the correct GA genotype, whereas the incorrect GG genotype would lead to the prediction of a “dark-black” phenotype. For individual C from GEDNAP 65, a “pale to intermediate” skin colour would be predicted with the correct AA genotype, whereas the incorrect AG genotype would lead to the prediction of a “pale” phenotype. However, it should be kept in mind that the predicted phenotype depends on the combination of all alleles detected for the respective individual, thus, the impact of individual genotyping errors on phenotyping results may be larger in other cases.

**Table 5:** Predicted probabilities for individuals with discrepant genotyping results for each of the five skin type categories obtained with the Hirisplex-S-webtool [16]

Prof. test sample	SNP	Genotype input	Visual Phenotype	Prediction probability (Hirisplex-S webtool)					
				Very pale	Pale	Inter-mediate	Dark	Dark- black	
GEDNAP 65 ind. B	rs10756819 (BNC2)	GA* GG	Dark/dark-black	0.000 0.000	0.000 0.000	0.000 0.000	0.463 0.293	0.536 0.706	
GEDNAP 65 ind. A	rs1126809 (TYR)	AA* AG	Intermediate	0.020 0.059	0.595 0.573	0.372 0.358	0.012 0.010	0.000 0.000	
GEDNAP 65 ind. C	rs1126809 (TYR)	AA* AG	Pale	0.105 0.262	0.611 0.504	0.283 0.233	0.001 0.001	0.000 0.000	
TrACE 2023 ext. ind. 2	rs1470608 (OCA2)	GT* GG	Dark-black	0.000 0.000	0.000 0.000	0.000 0.000	0.009 0.022	0.991 0.978	
TrACE 2024 ext. ind. 1	rs1126809 (TYR)	AA* AG	Intermediate	0.049 0.016	0.154 0.157	0.708 0.724	0.089 0.102	0.000 0.000	

*correct genotype

Overall, it should be kept in mind that the majority of samples used to train the HIrisPlex-S phenotype prediction model [16] were genotyped using the SNaPshot-based method on capillary electrophoresis with the original HIrisplex-S primer sets. It thus remains questionable whether the use of methods allowing to obtain more correct genotyping results (e.g. by being able to unambiguously differentiate between amplicons generated from the TYR gene and the TYRL pseudogene with MPS-based methods or being able to mitigate the impact of primer binding region mutations by using adapted primer sequences) generally increases phenotype prediction accuracy. This effect might be more pronounced for variants more common in non-European populations, which are underrepresented in the HIrisPlex-S training dataset and for which a genotyping error in the training dataset can thus be expected to have a more severe impact on model prediction performance.

To be able to assess the impact of potential errors in the HIrisplex-S training data set (arising from genotyping biases due to primer binding site mutations, the overlap of SBE primers or off-target amplification of the TYRL pseudogene), the MPS-based verification and/or extension of the training dataset would be desirable.

In case of ambiguous or potentially divergent results, obtaining phenotype predictions with different input options for the affected SNP to assess the impact on phenotyping results is recommended. However, for users of community or commercial primer panels, it is not always possible to assess whether and how these differ from the original HIrisPlex-S primer panel and thus might potentially produce divergent genotyping results (e.g. in case of primer binding site mutations). For the sake of genotyping consistency, we thus highly recommend that custom or commercially designed primer sequences should be made publicly available or at least that deviations from primer sequences used to generate the training data set should clearly be communicated.

## Conclusion

In recent years, the HIrisplex-S-system has become widely used for the purpose of forensic DNA phenotyping, but different laboratory methods (SNaPshot™-based or MPS-based) with a range of self-designed or commercially produced primer panels are employed for genotyping.

The GEDNAP and TrACE collaborative exercises, for which samples from the same individuals were analysed by a larger number of laboratories each employing their own methodology, revealed that laboratories mostly obtained consistent genotyping results, however, systematic discrepancies were observed for individual SNP targets. An in-depth analysis revealed primer binding site mutations and the potential for off-target amplification due to high sequence similarities to other genomic regions as causes of discrepant genotyping results.

While MPS-based methods tended to better overcome these issues (most likely due the use of adjusted primer sequences and the possibility of assessing the entire amplicon sequence), the benefit of these improvements remains questionable due the use of SNaPshot-based methods for the generation of the prediction model’s training data. Additionally, the use of proprietary primer sequences in most MPS-based approaches complicates a direct comparison of results. While the impact on phenotyping results was minor to negligible in all cases reported here, the issues uncovered by this in-depth analysis of discrepant genotyping results may provide a basis for improvements towards more consistent methods in the future.

## Supplementary Information

Below is the link to the electronic supplementary material.

Supplementary Material 1

Supplementary Material 2

Supplementary Material 3

## Data Availability

All data can be found within the manuscript and supplementary files.
