# Supplementary material for: Technical reliability of genotyping SNPs for forensic DNA phenotyping using SNaPshot- and MPS-based assays
Source: Int J Legal Med. 2026 Jan 8;140(3):1273–85. doi: 10.1007/s00414-025-03709-6 (PMC13161349; doi:10.1007/s00414-025-03709-6)
Supplement: Supplementary file 2 — Supplementary Material 2 [file 414_2025_3709_MOESM2_ESM.docx]

**Supplementary File 2:**

Screenshots showing PCR amplicons visualised in the NCBI Sequence Viewer. The variation track shows all RefSNPs in dbSNP b157 v2 and SNPs from the 1000 Genomes Phase 3, while the table lists all variants with frequency information from the 1000 Genomes Project, Phase 3 (global MAF). Variants in primer binding regions with an MAF >0.01 are indicated in Supplementary Tables 1-3.

**Assay 01**

**Set 1**


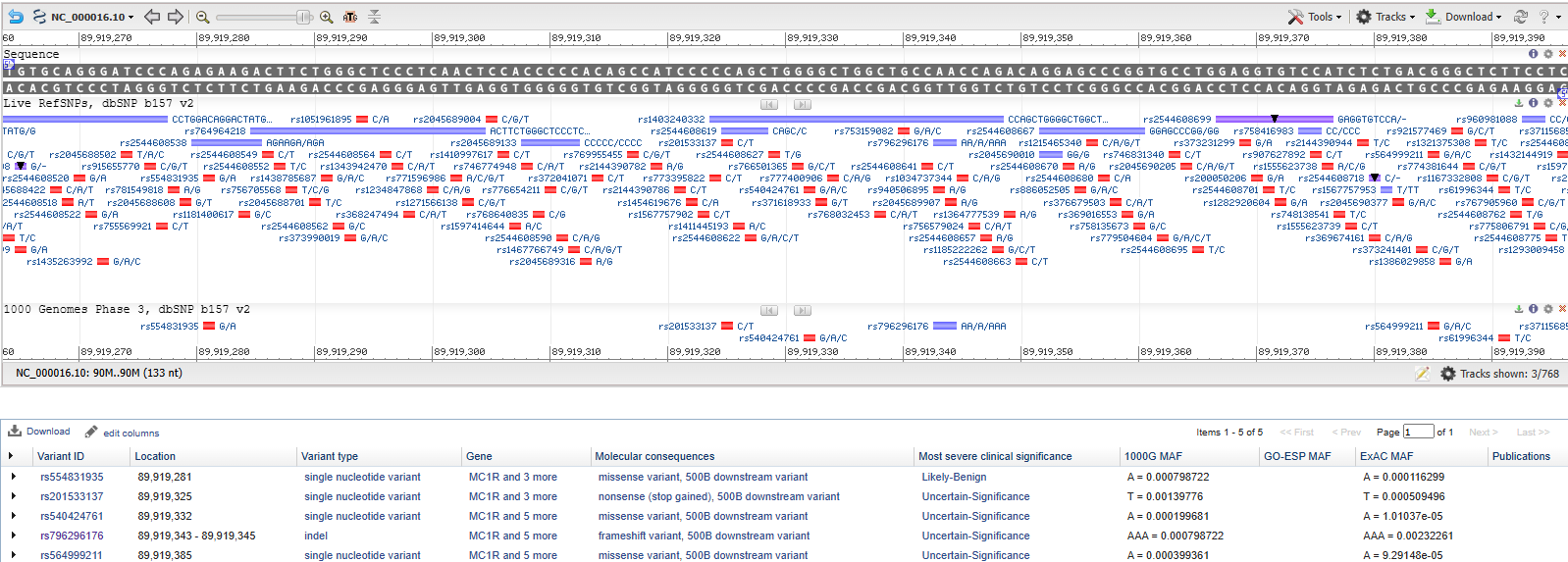


**Set 2**


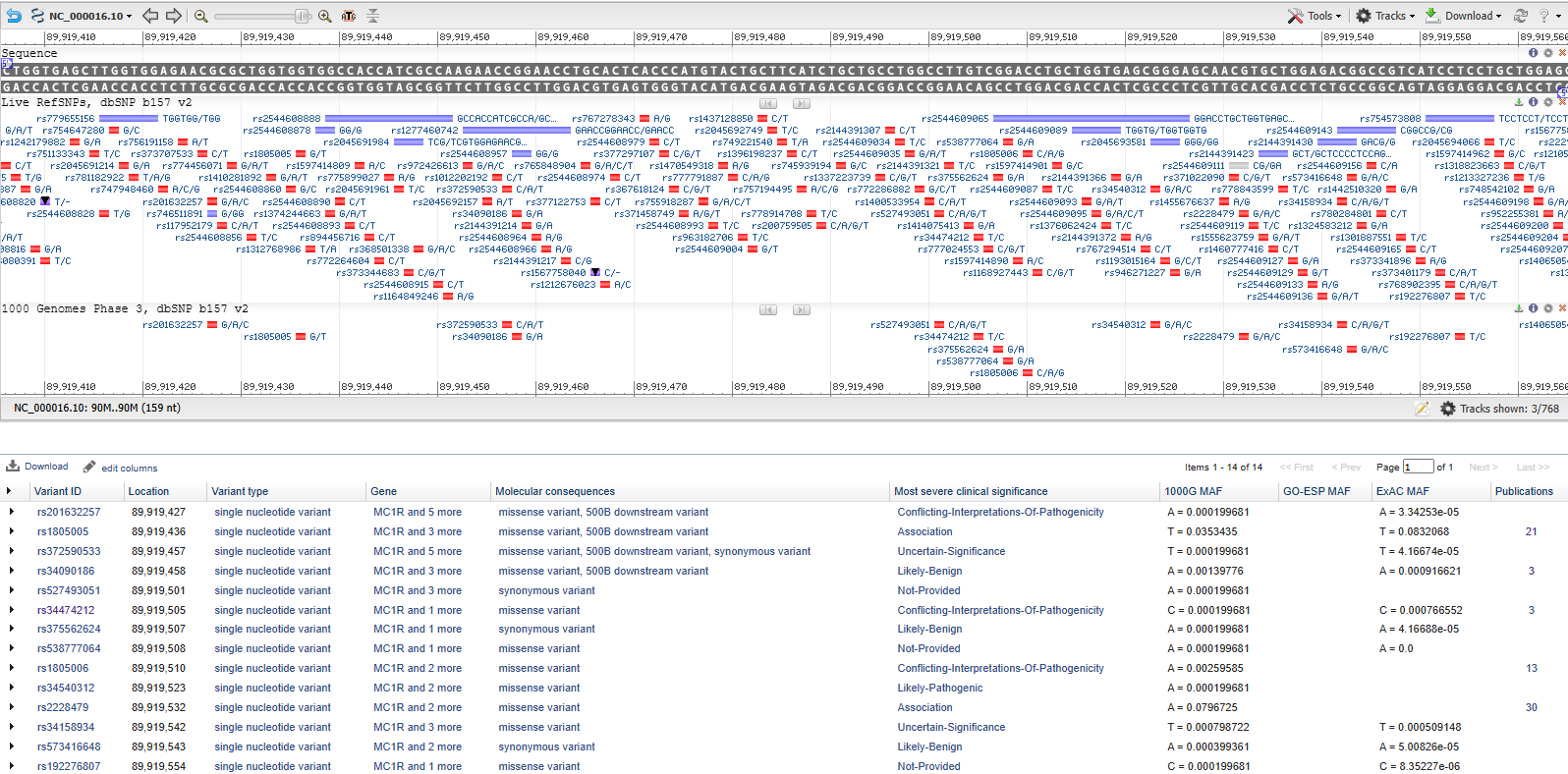


**Set 3**


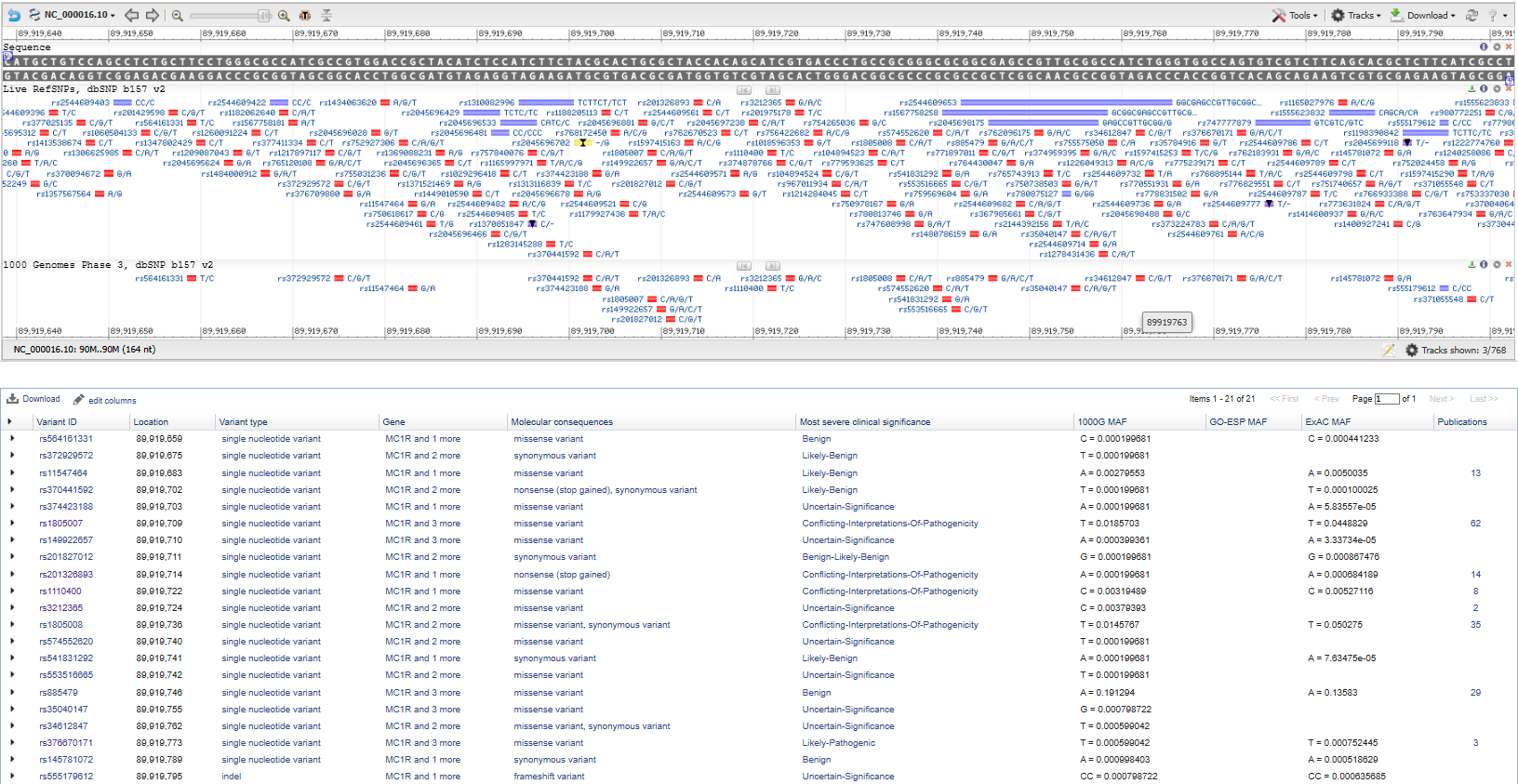


**Set 4**

**
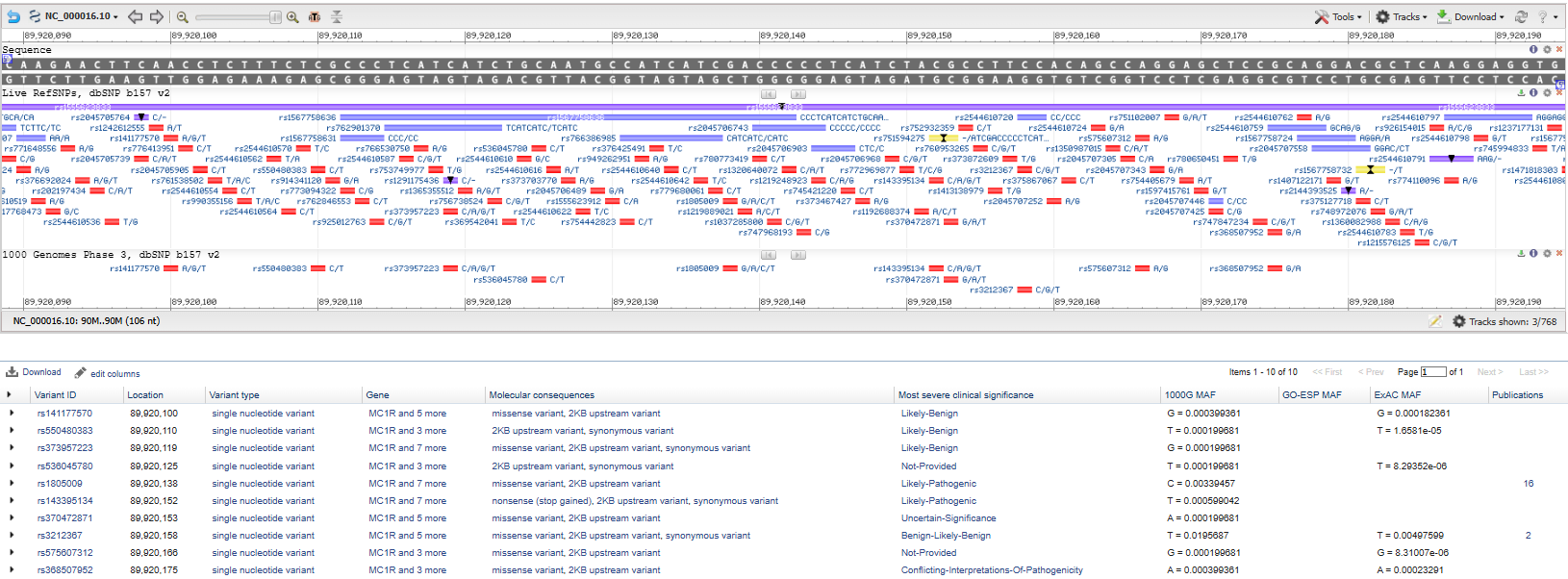
**

**Set 5**

**
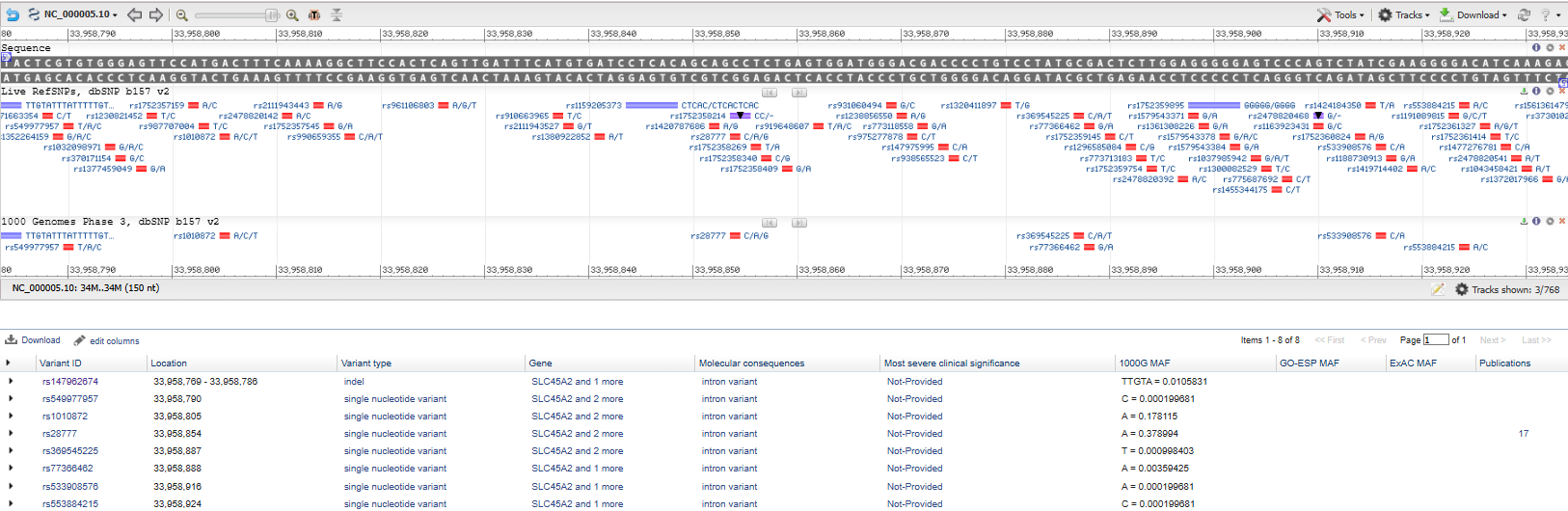
**

**Set 6**

**
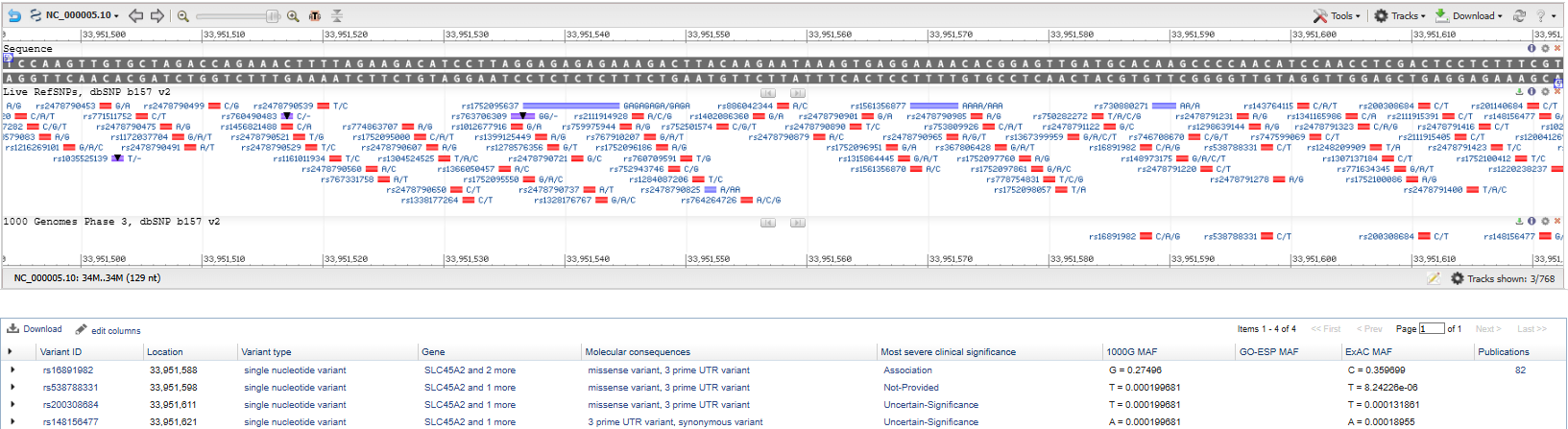
**

**Set 7**

**
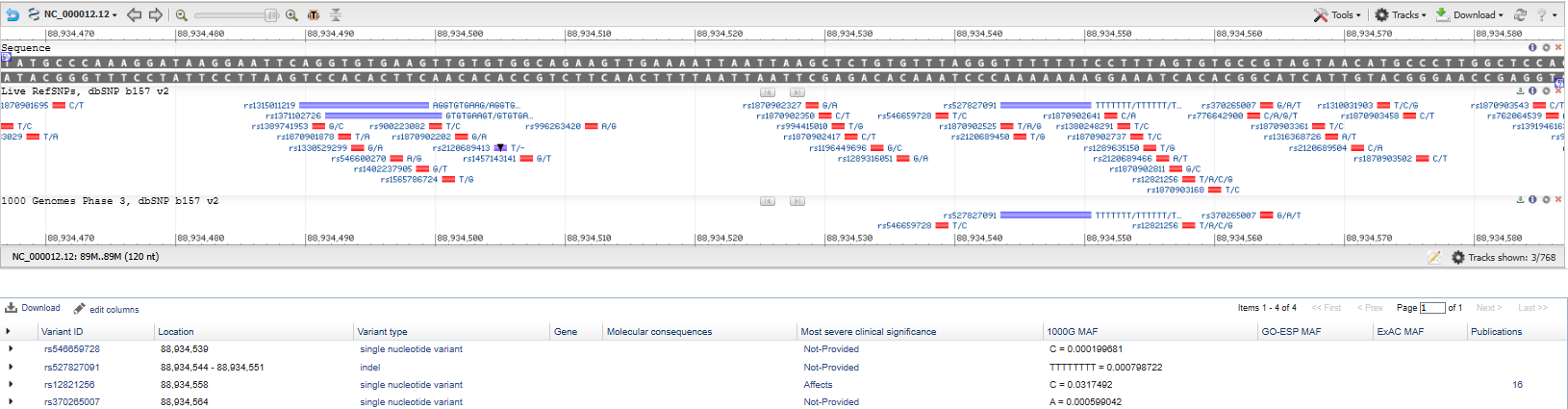
**

**Set 8**

**
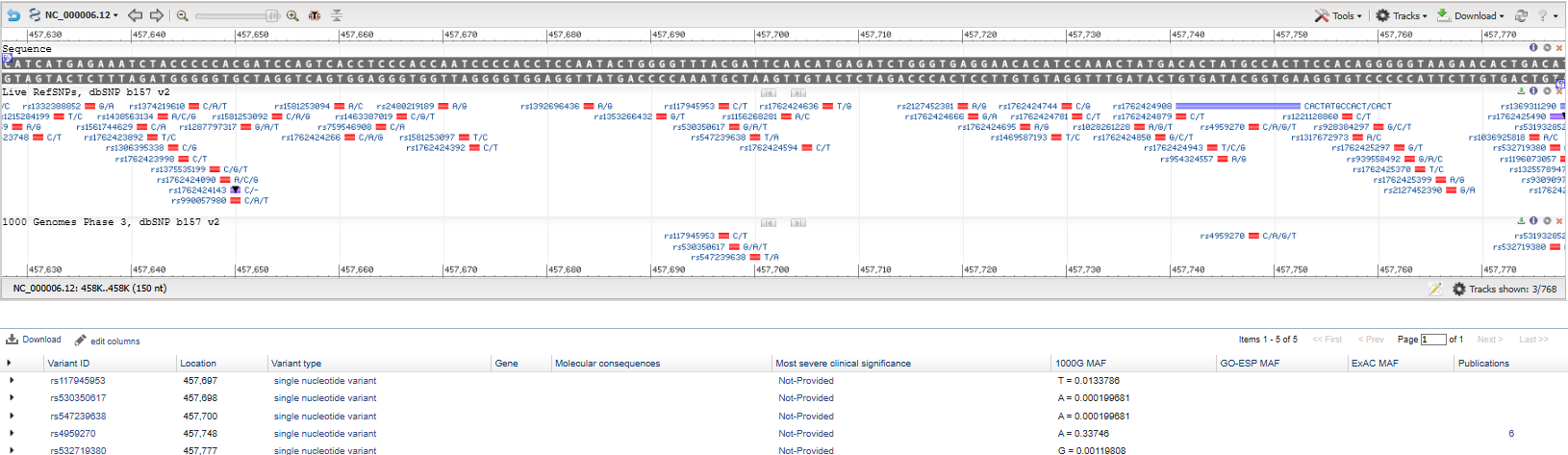
**

**Set 9**

**
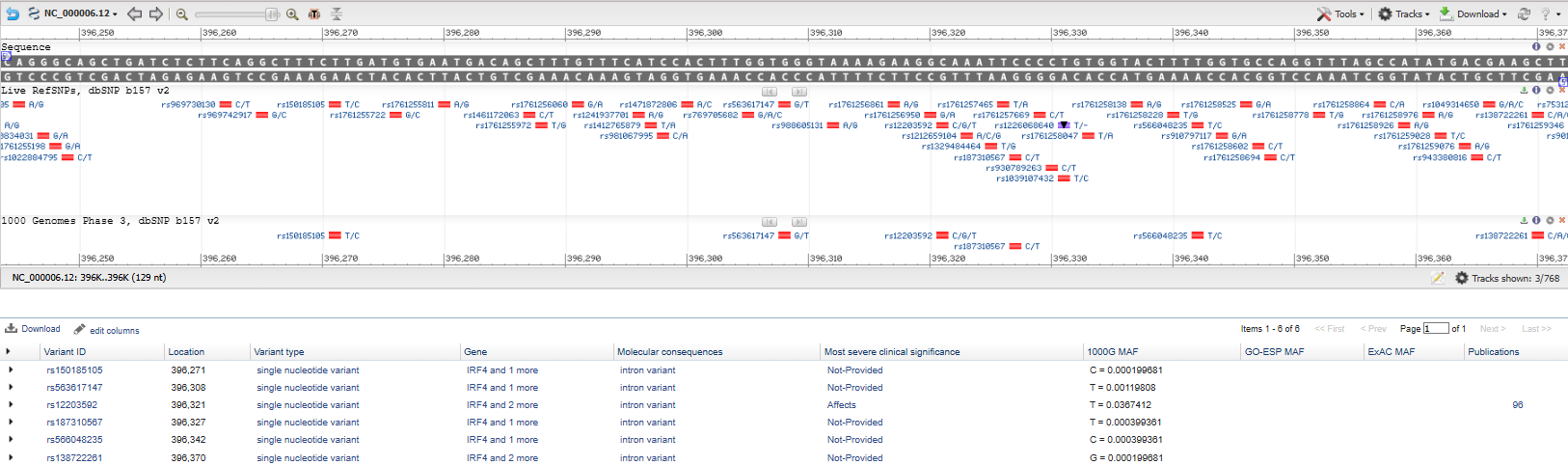
**

**Set 10**

**
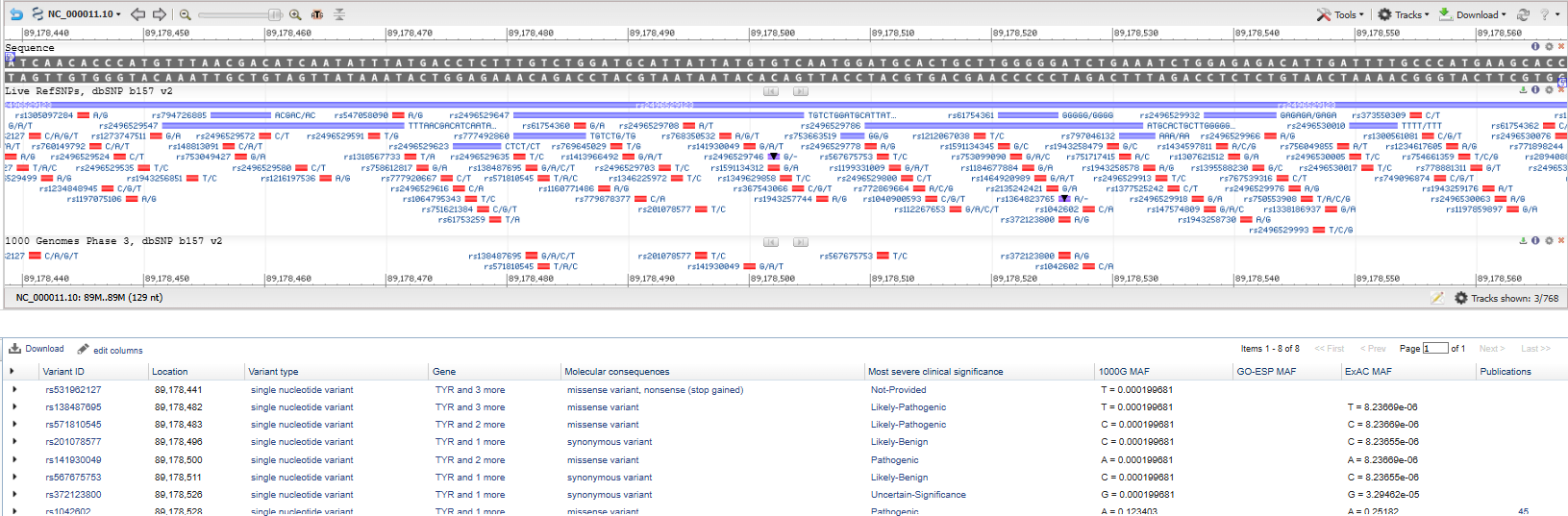
**

**Set 11**

**
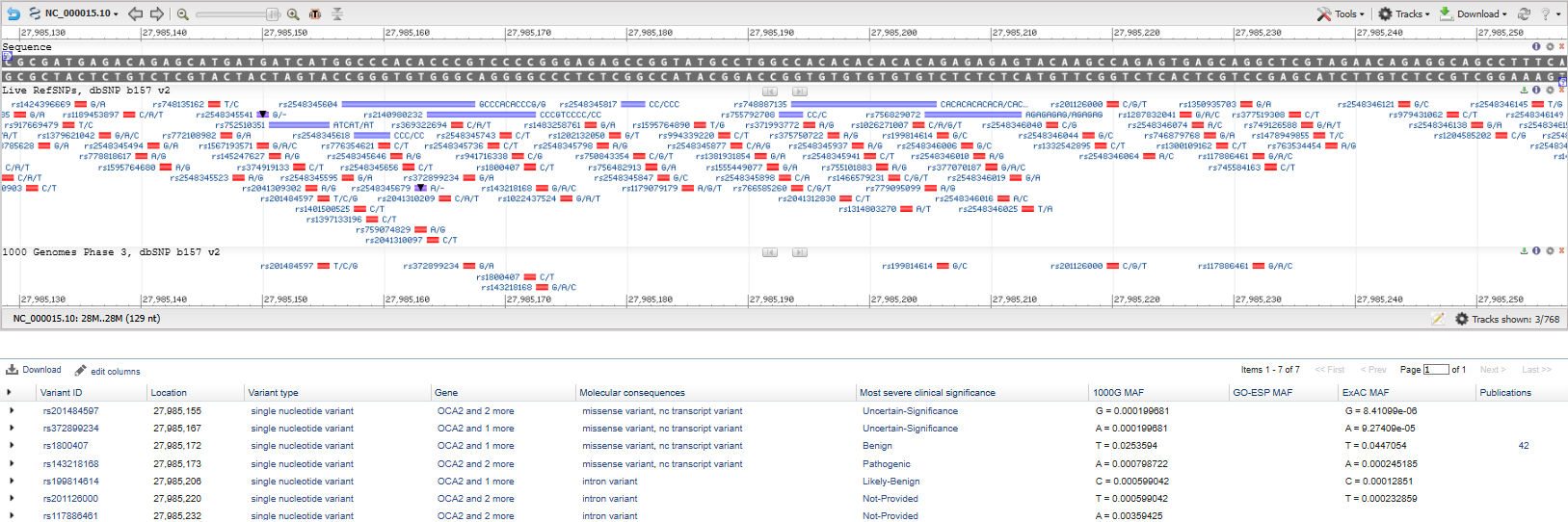
**

**Set 12**

**
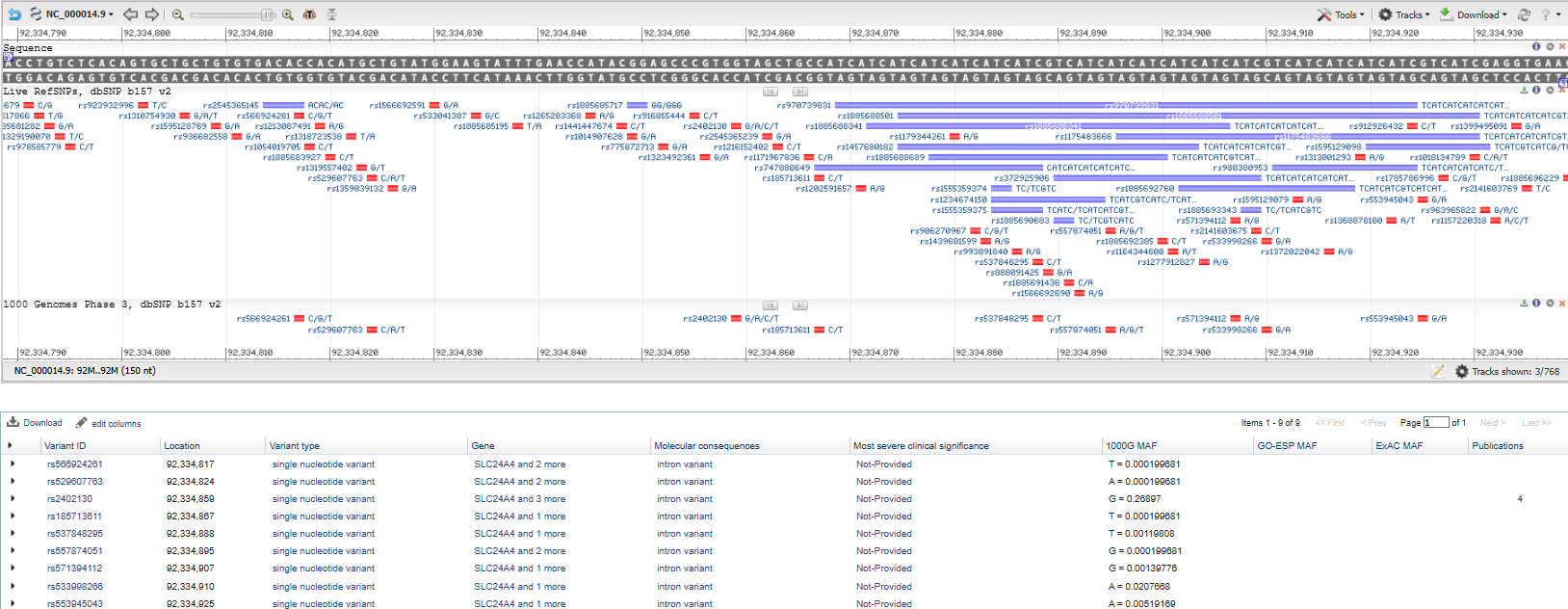
**

**Set 13**

**
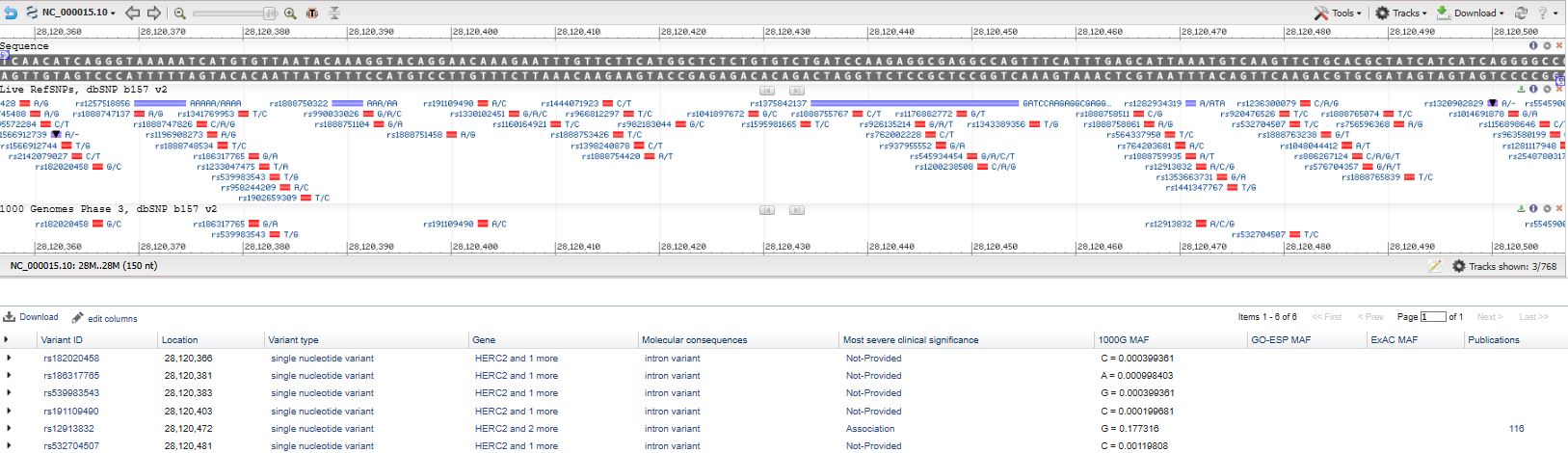
**

**Set 14**

**
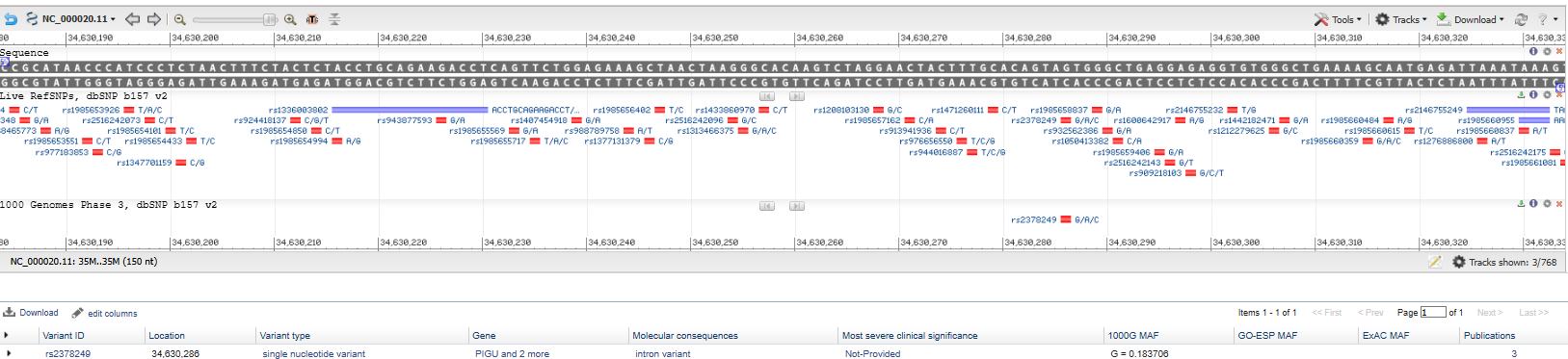
**

**Set 15**

**
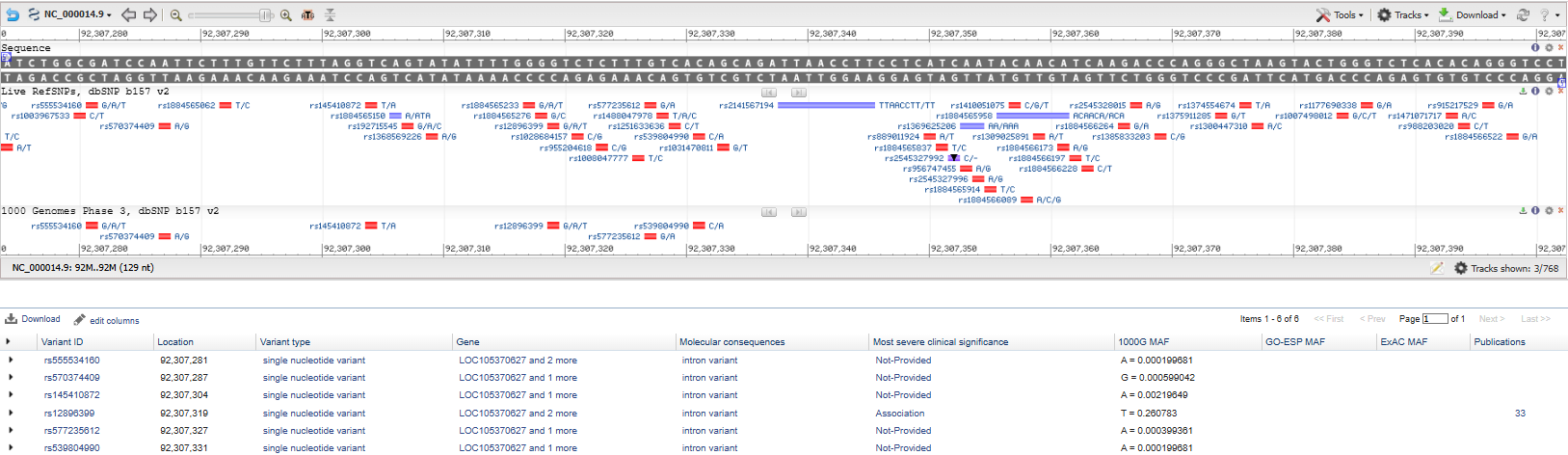
**

**Set 16**

**
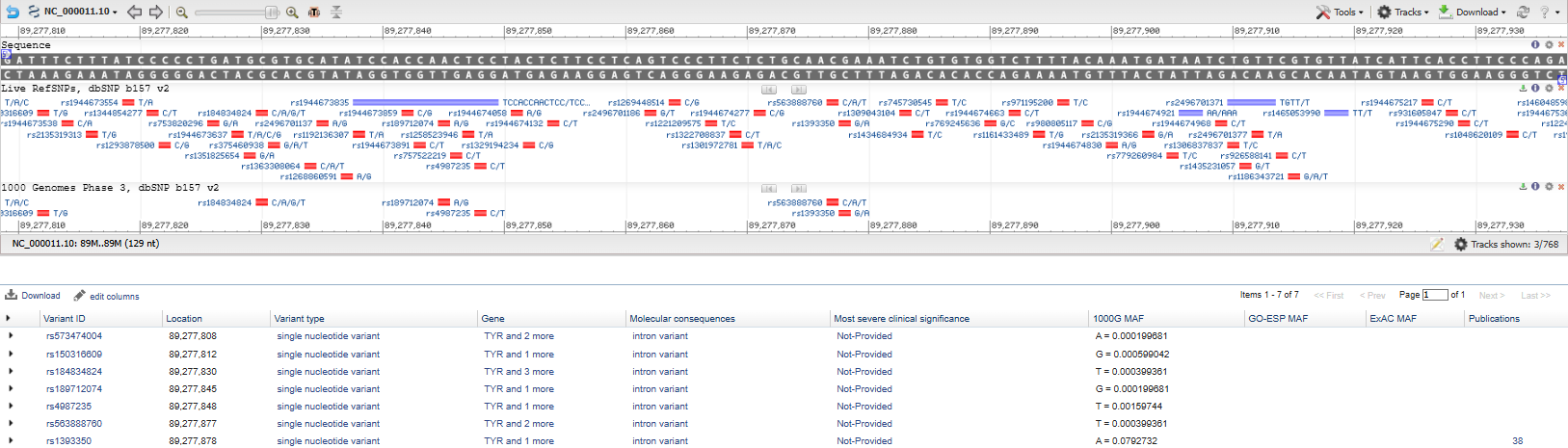
**

**Set 17**

**
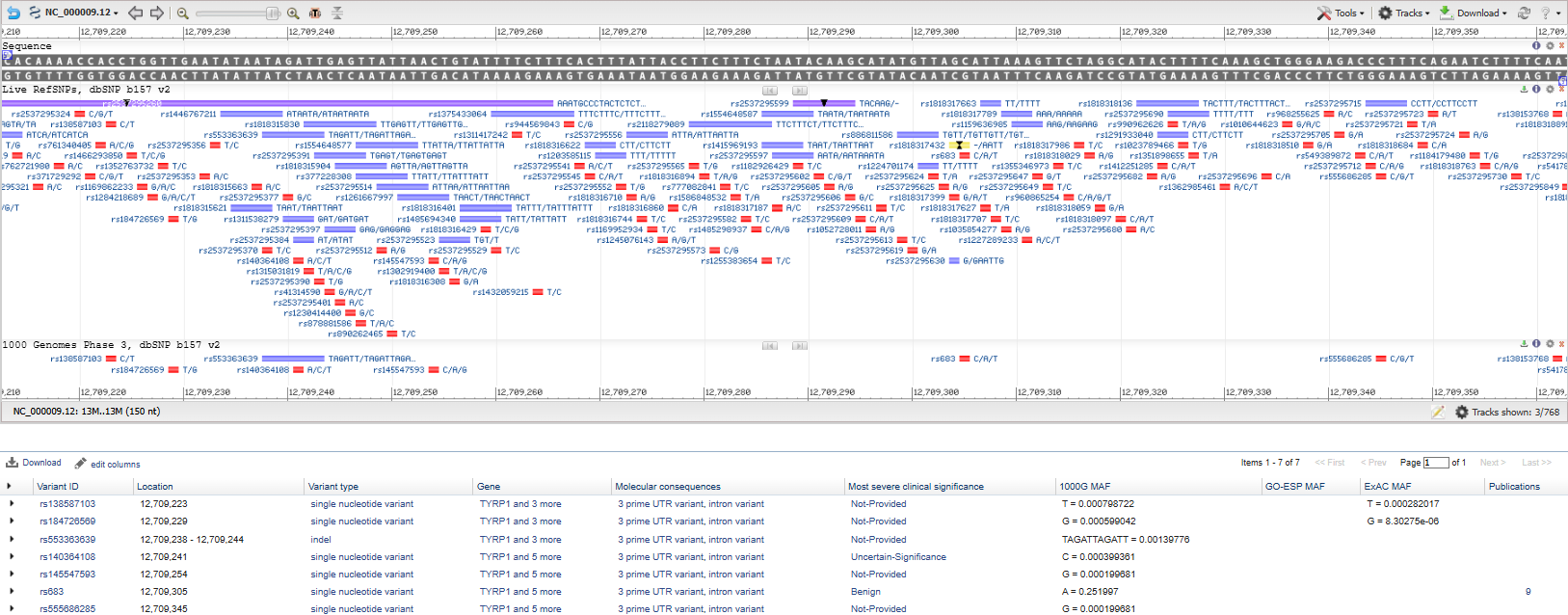
**

**Assay 02:**

**Set 1**

**
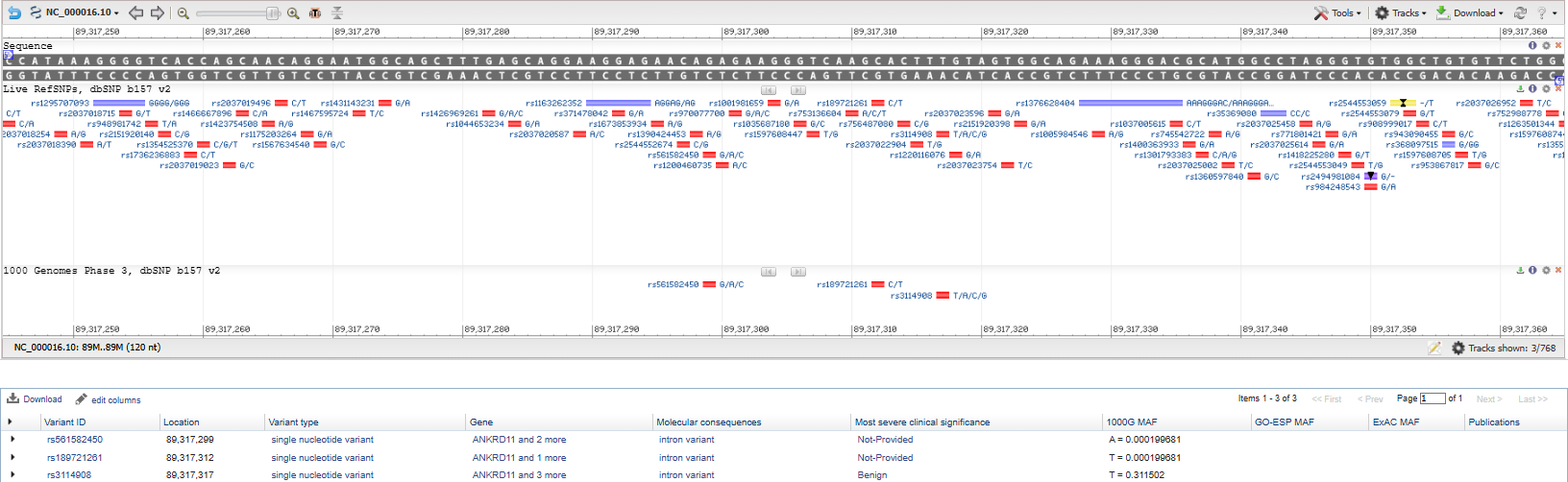
**

**Set 2**

**
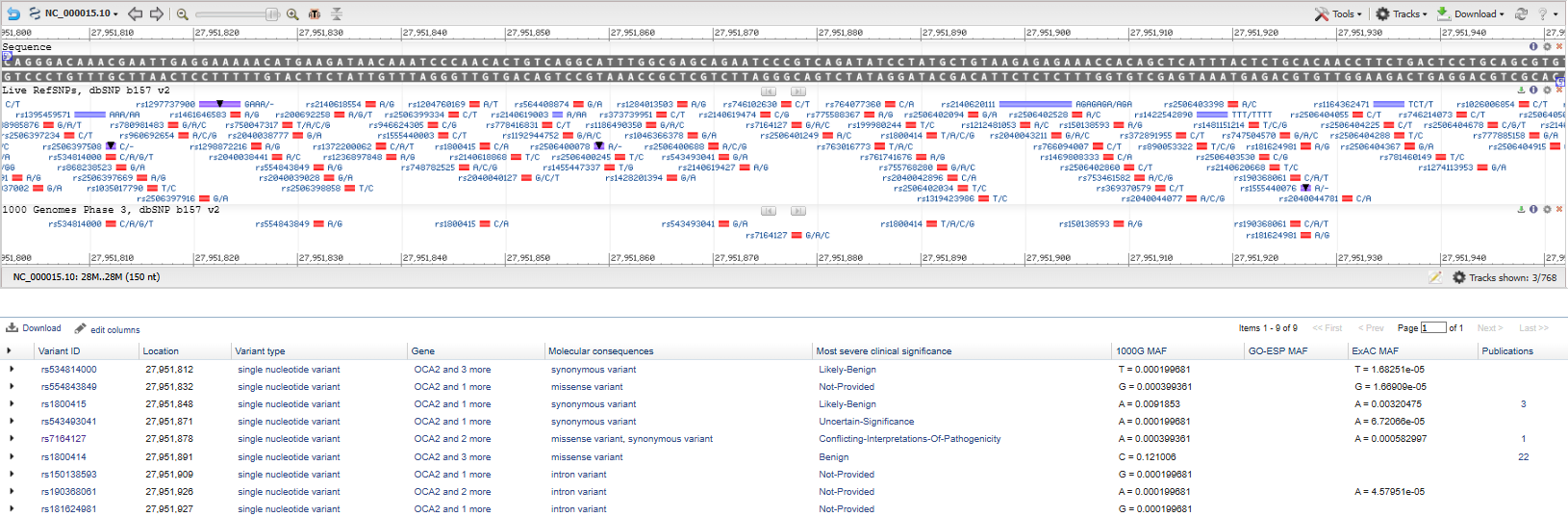
**

**Set 3**

**
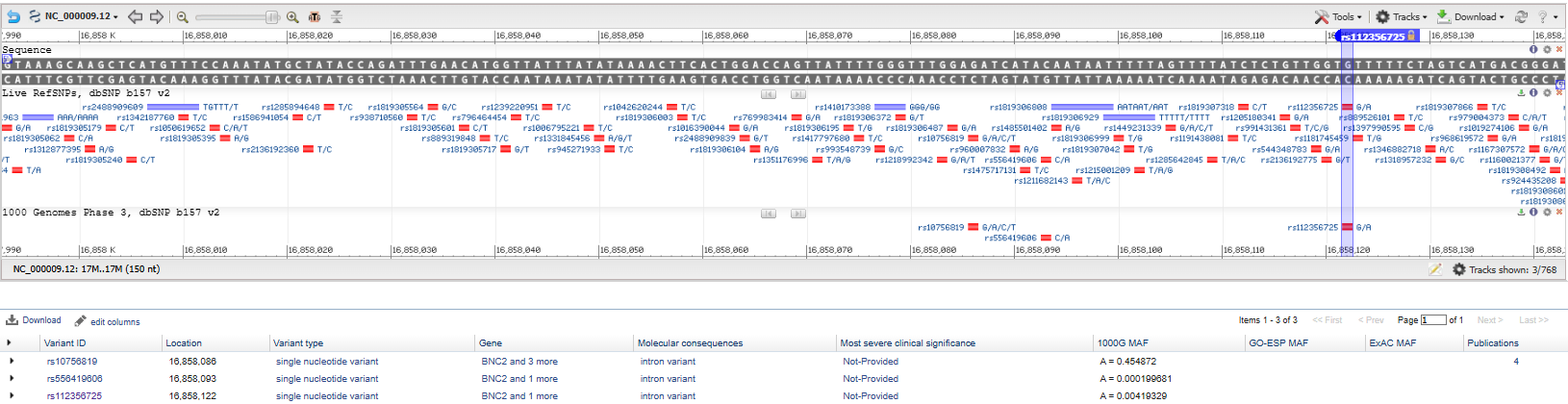
**

**Set 4**

**
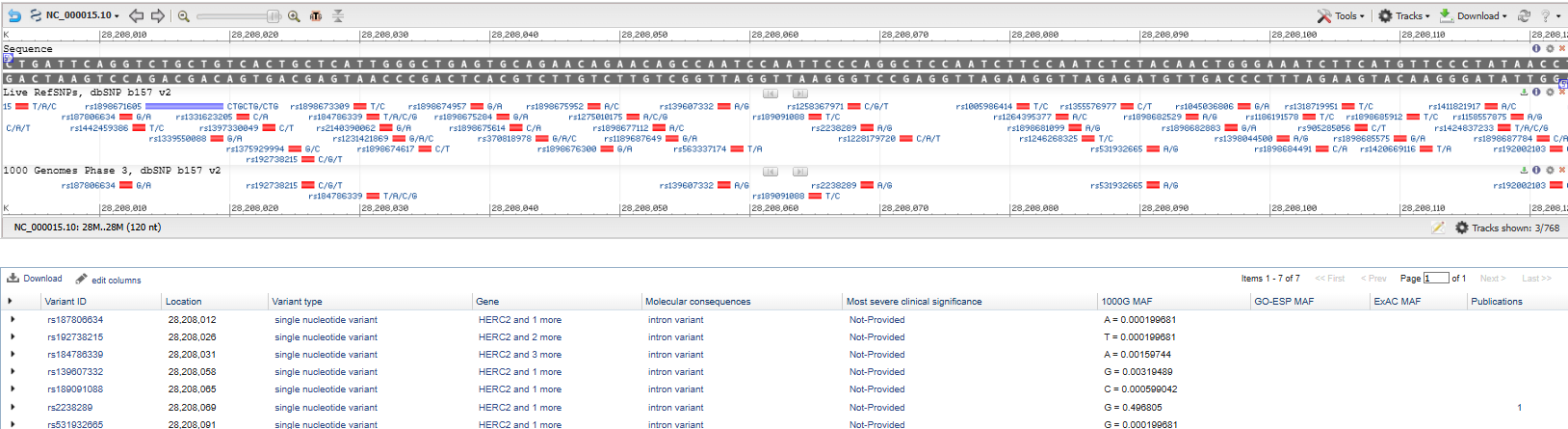
**

**Set 5**

**
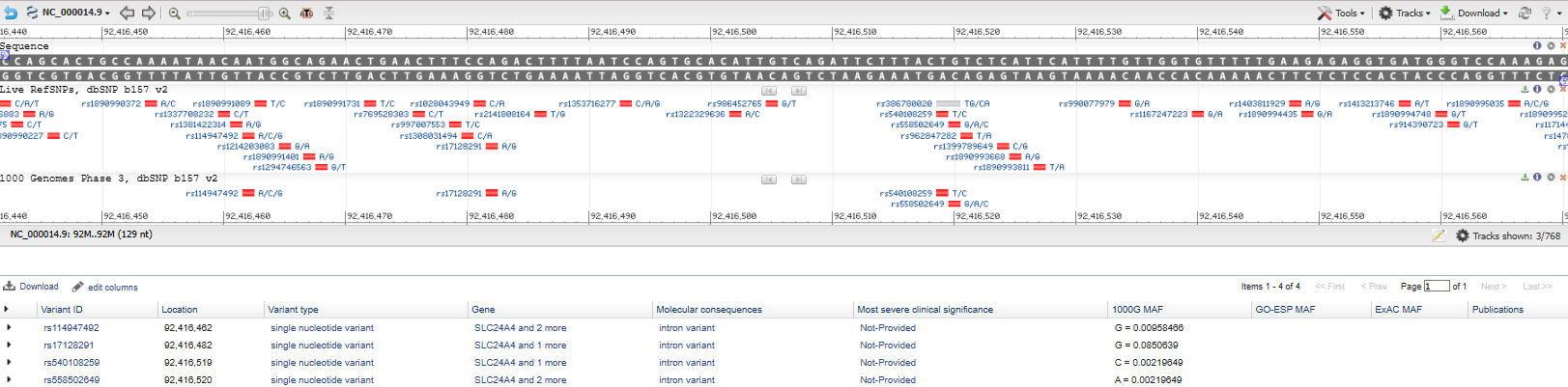
**

**Set 6**

**
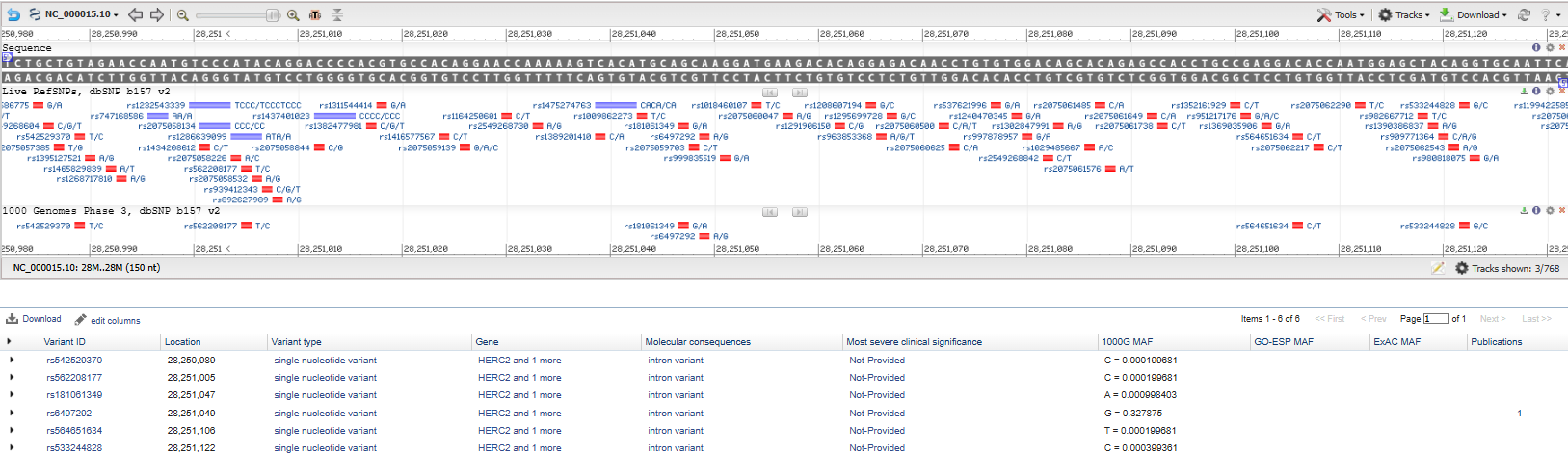
**

**Set 7**

**
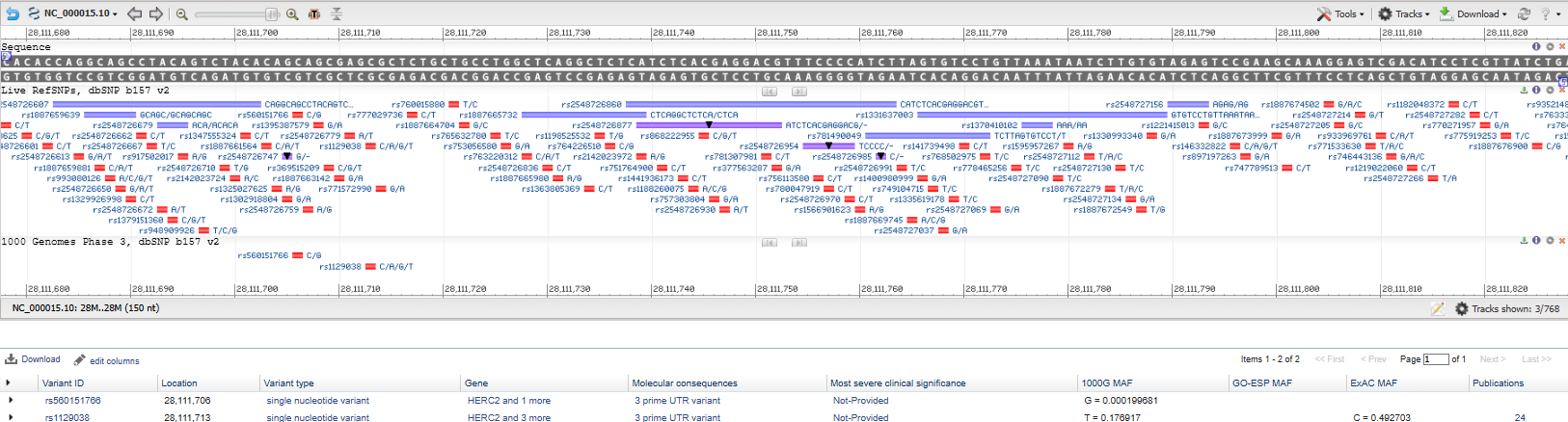
**

**Set 8**

**
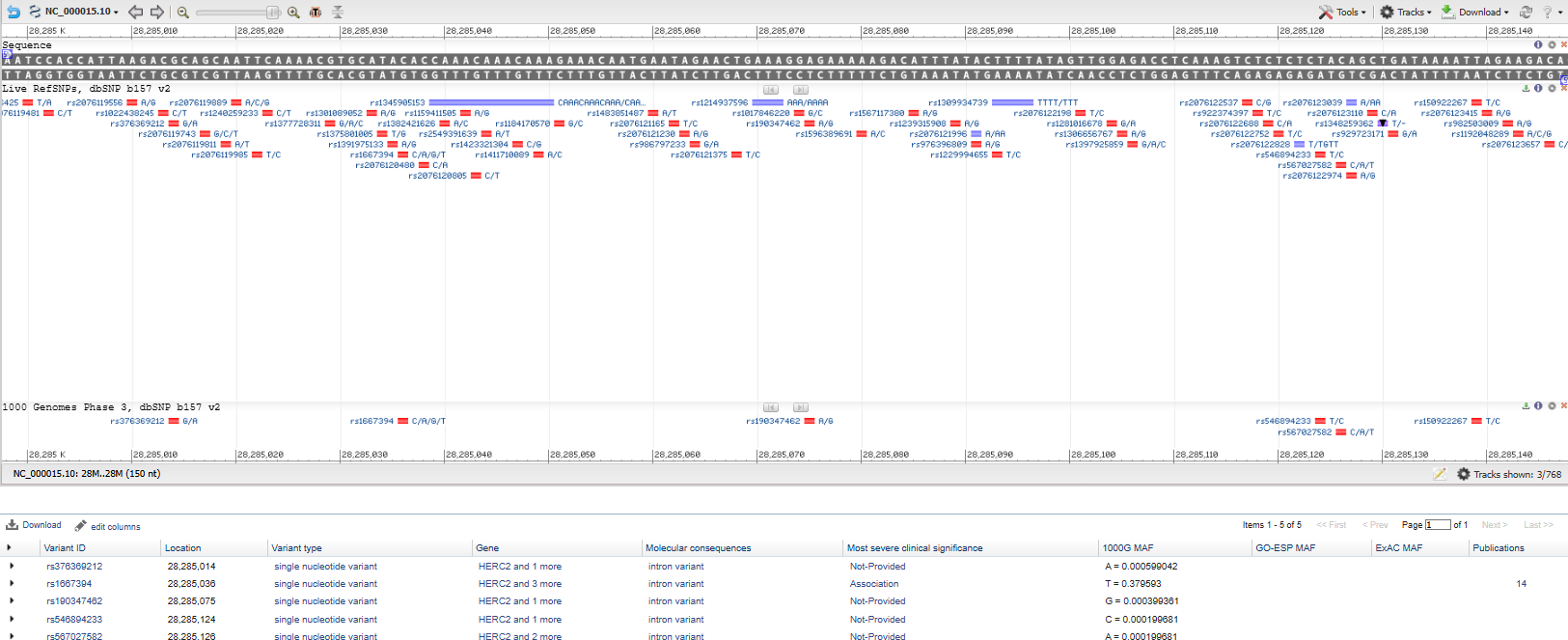
**

**Set 9**

**
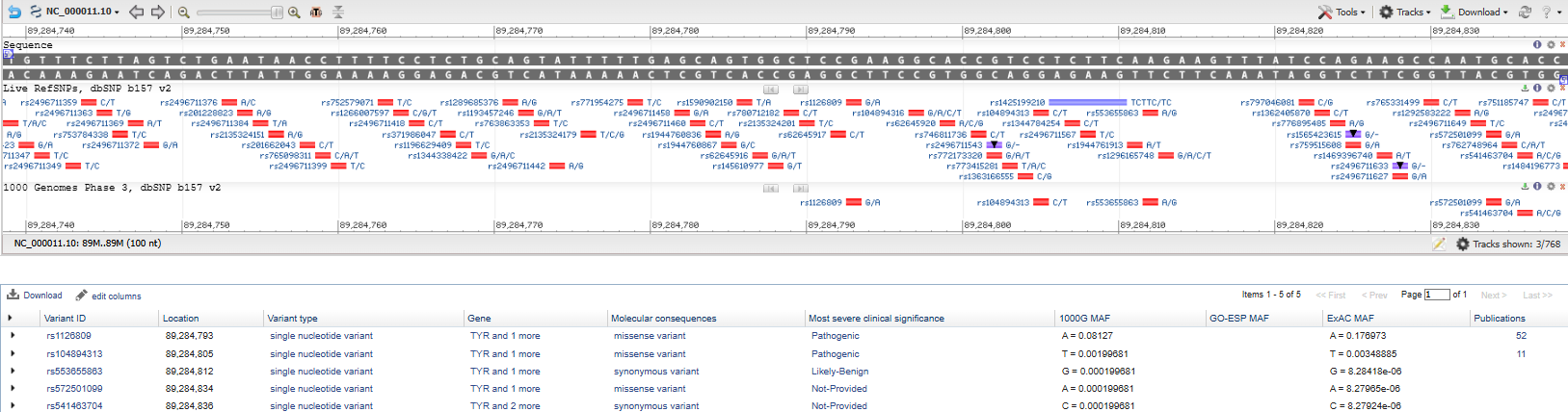
**

**Set 10**

**
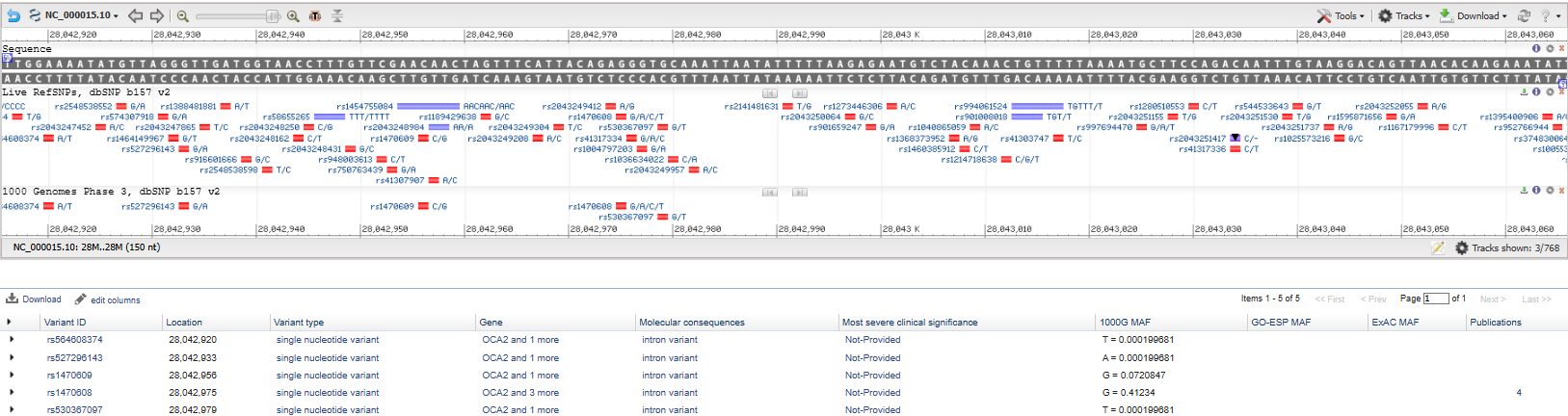
**

**Set 11**

**
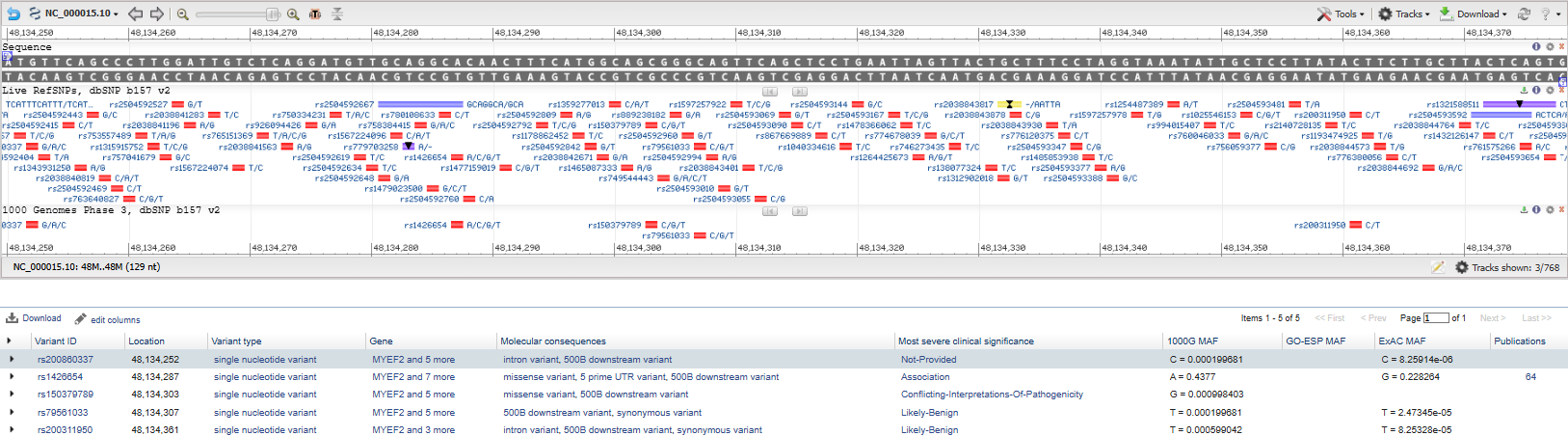
**

**Set 12**

**
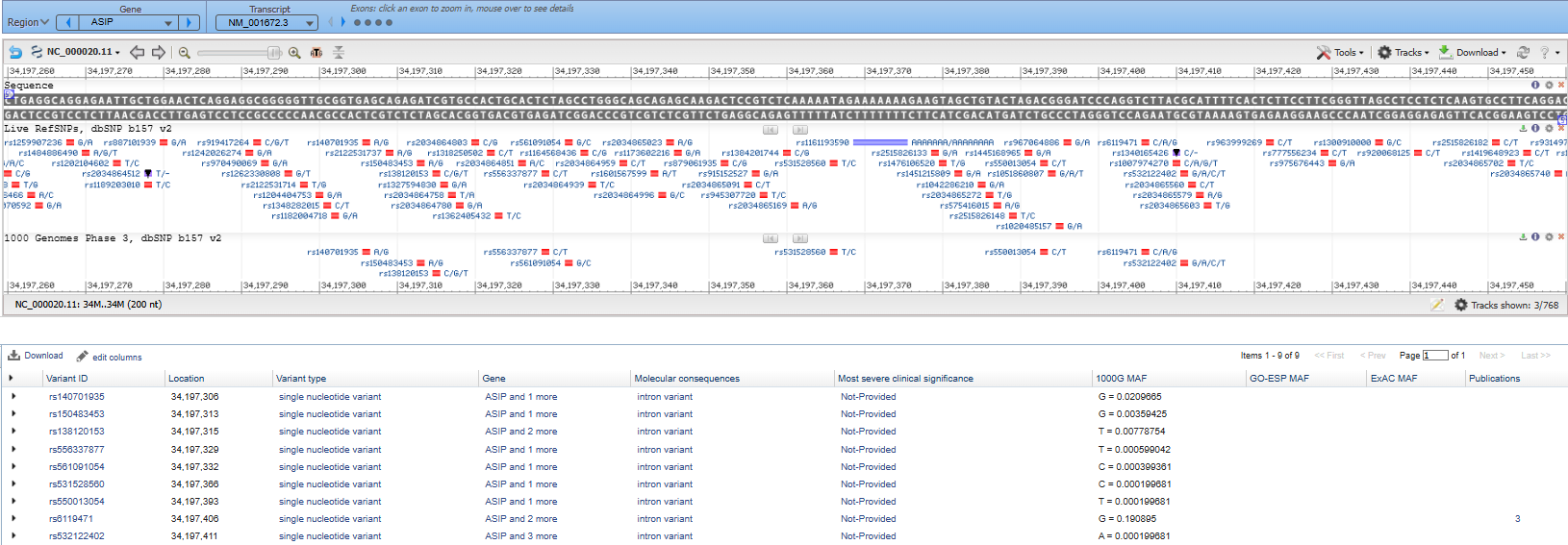
**

**Set 13**

**
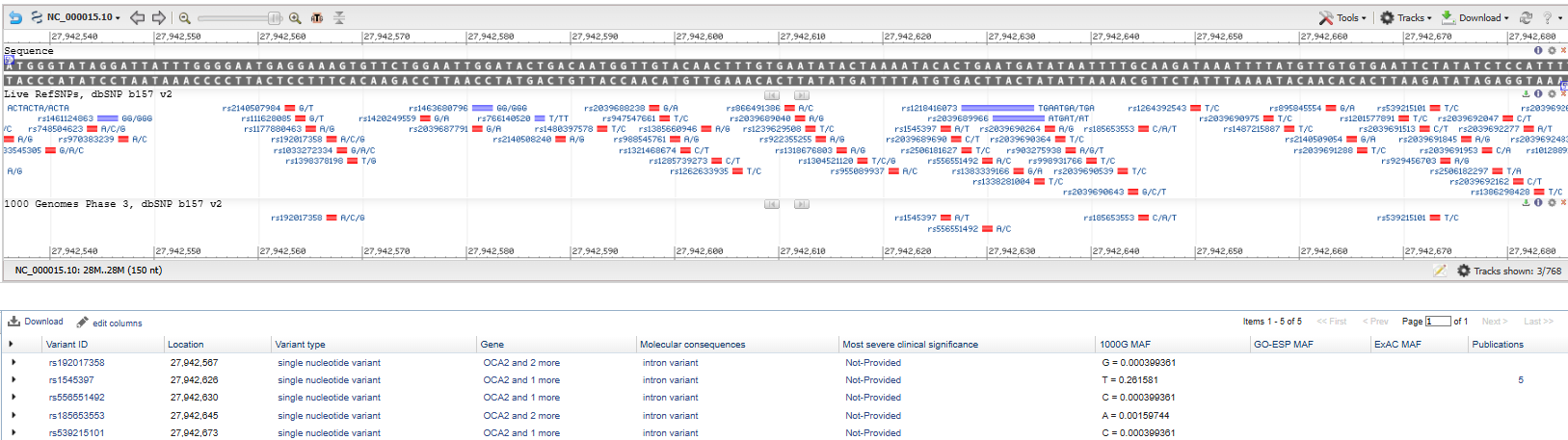
**

**Set 14**

**
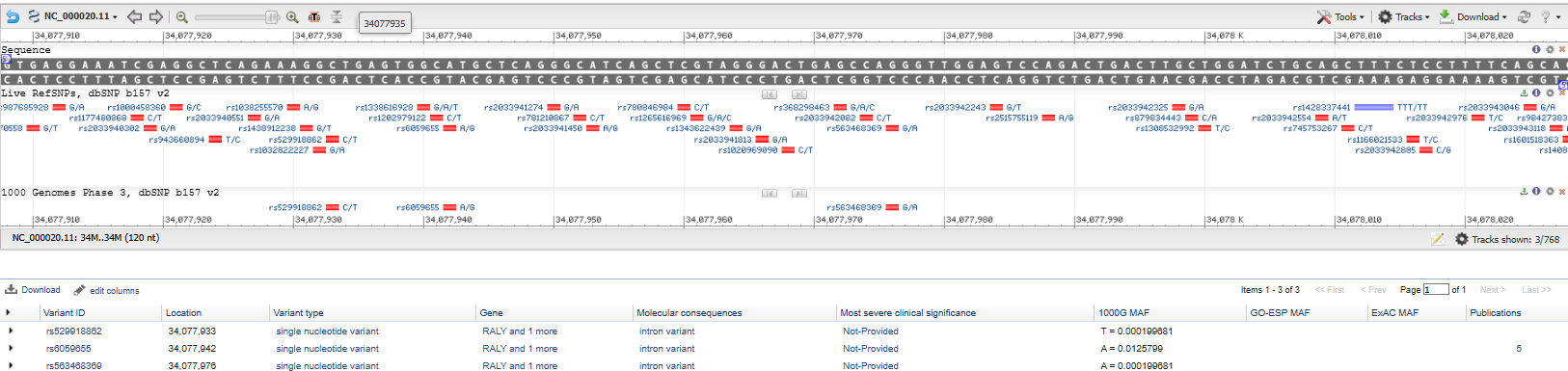
**

**Set 15**

**
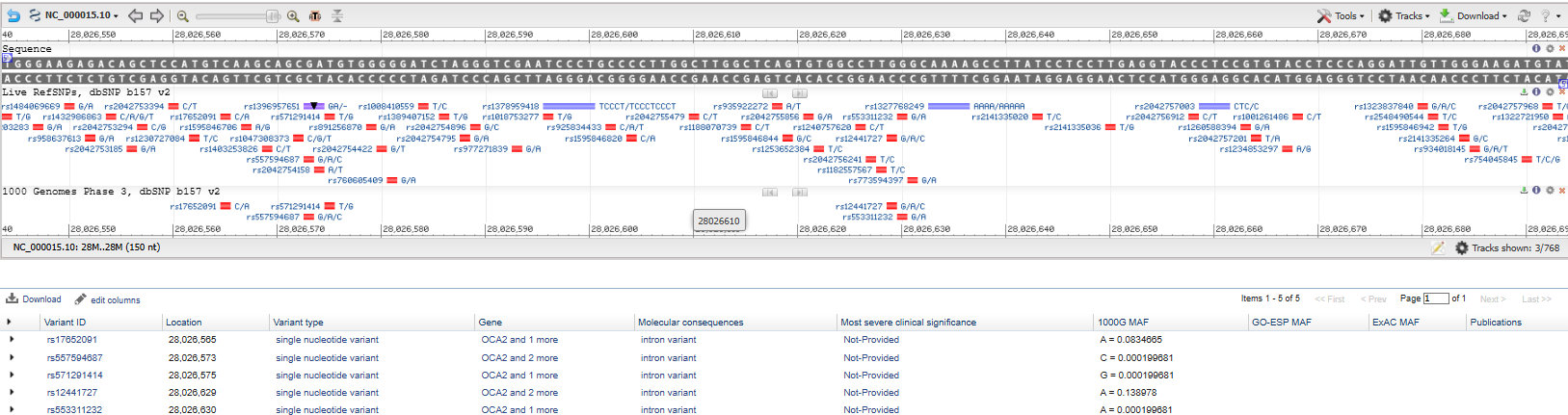
**

**Set 16**

**
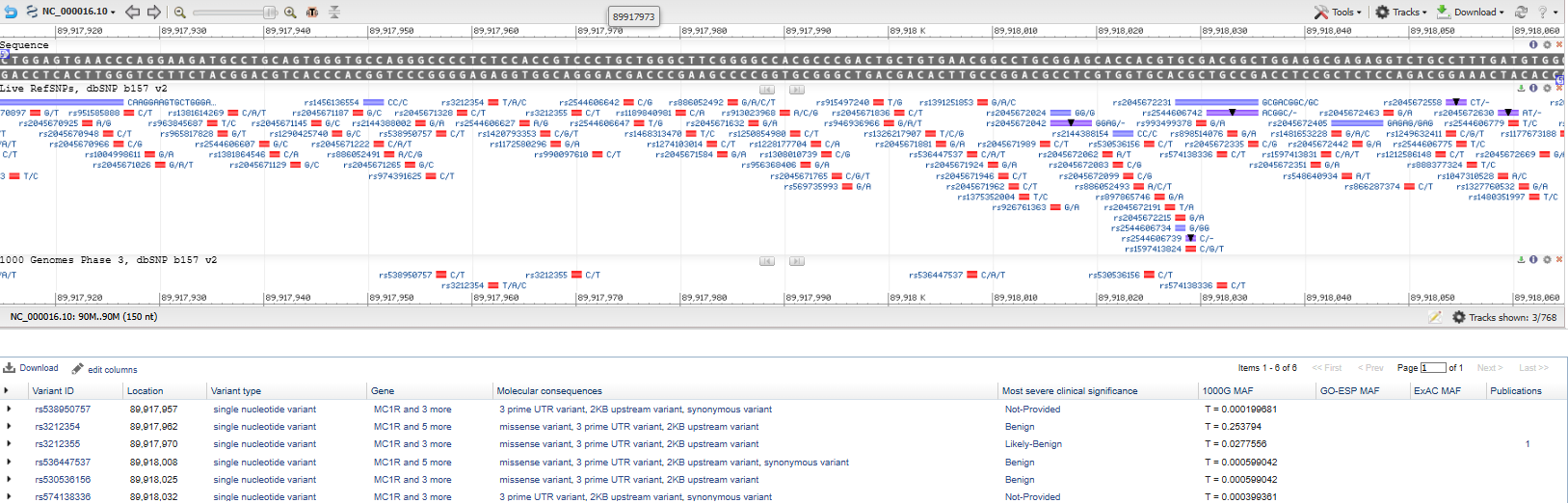
**

**Set 17**

**
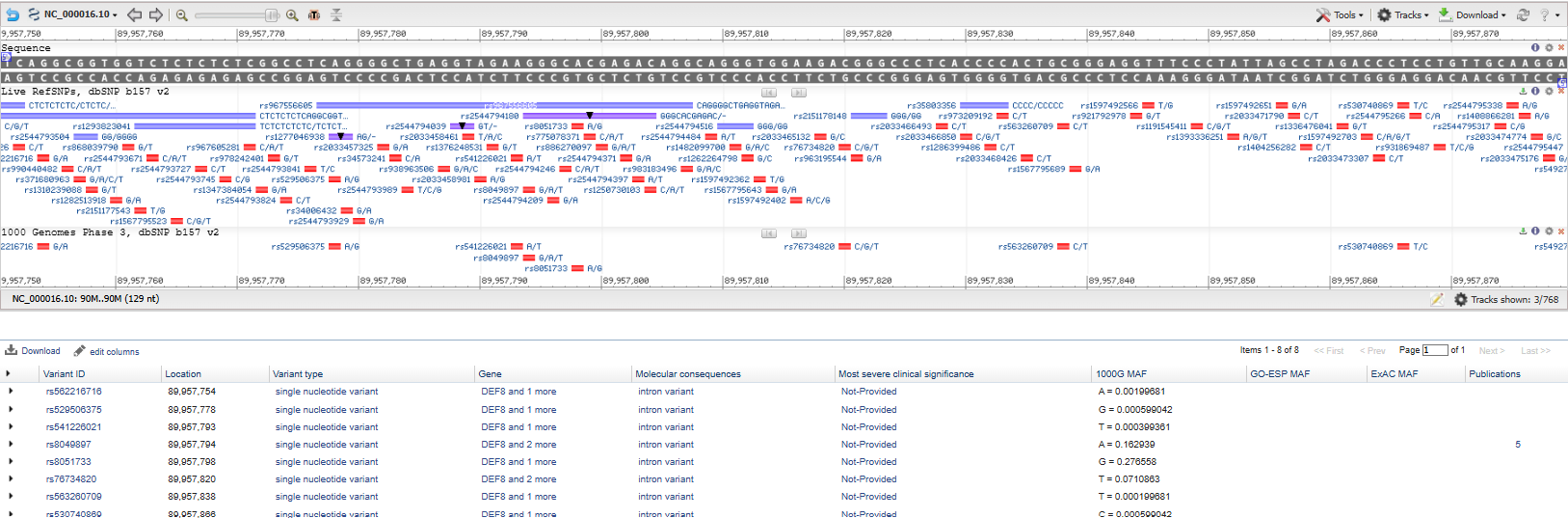
**
