# Supplementary material for: Technical reliability of genotyping SNPs for forensic DNA phenotyping using SNaPshot- and MPS-based assays
Source: Int J Legal Med. 2026 Jan 8;140(3):1273–85. doi: 10.1007/s00414-025-03709-6 (PMC13161349; doi:10.1007/s00414-025-03709-6)
Supplement: Supplementary file 3 — Supplementary Material 3 [file 414_2025_3709_MOESM3_ESM.docx]

**Supplementary File 1:**

Sequences of „high-risk“ off-target PCR products (<1000 bp and maximum of 1 mismatch per primer) in FASTA format. Binding regions of the corresponding PCR primers are underlined (with mismatches not underlined and highlighted in grey). Binding regions of the corresponding SBE primers are highlighted in blue (with mismatches shown in red) and the nucleotide detected by the SNaPshot assay is highlighted in orange.

**Assay 01:**

None

**Assay 02:**

**Set 06: rs6497292**

Target PCR product:

>ref|NC_000015.10|:28250982-28251131 Homo sapiens chromosome 15, GRCh38.p14 Primary Assembly

TCTGCTGTAGAACCAATGTCCCATACAGGACCCCACGTGCCACAGGAACCAAAAAGTCACATGCAGCAAGGATGAAGACACAGGAGACAACCTGTGTGGACAGCACAGAGCCACCTGCCGAGGACACCAATGGAGCTACAGGTGCAATTC

„High-risk“ off-target PCR products:

1) Chr15:22476979-22477128

>ref|NC_000015.10|:22476979-22477128 Homo sapiens chromosome 15, GRCh38.p14 Primary Assembly

TCTGCTGTAGAACCAATGTCCCATACAGGACCCCACGTGCCACAGGAACCAAAAAGTCACACGCAGCGAA**−**GA**C**GAAGACACAGGAGACAACCTGTGTAGACAGCACAGAGCCACCTGCCCAGGACACCAATGGAGCCACAGGTGCAATTC


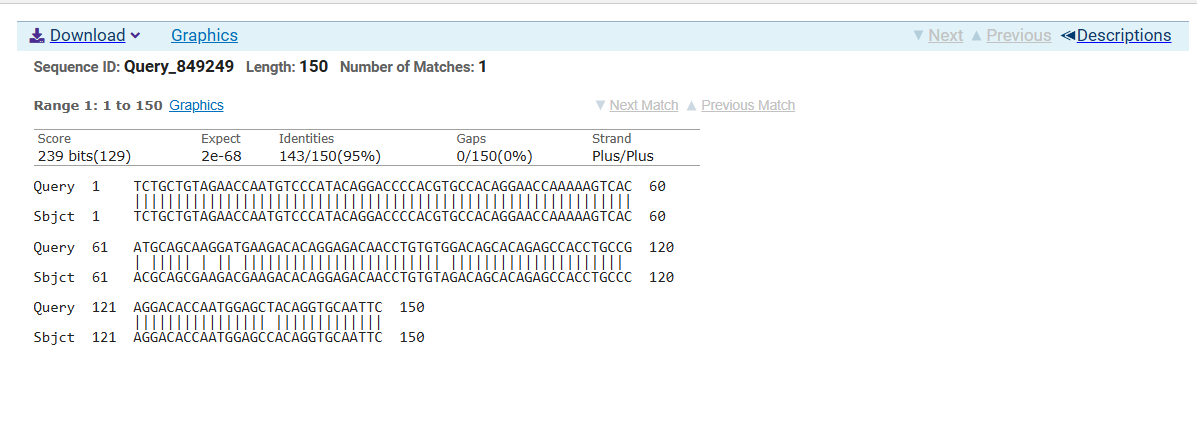


2 mismatches in SBE-primer

Detected allele: T

2) Chr15:23285821-23285970

>ref|NC_000015.10|:23285821-23285970 Homo sapiens chromosome 15, GRCh38.p14 Primary Assembly

TCTGCTGTAGAACCAATGTCCCATACAGGACCCCACGTGCCACAGGAACCAAAAAGTCACACGCAGCGAA**−**GA**C**GAAGACACAGGAGACAACCTGTGTGGACAGCACAGAGCCACCTGCCCAGGACACCAATGGAGCCACAGGTGCAATTC


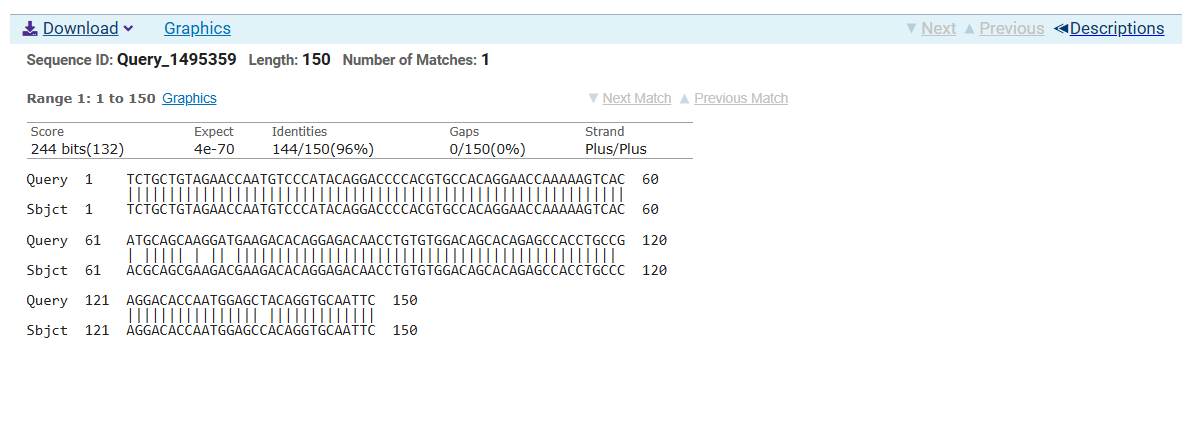


2 mismatches in SBE-primer

Detected allele: T

3) Chr15:28571551-28571700

>ref|NC_000015.10|:28571551-28571700 Homo sapiens chromosome 15, GRCh38.p14 Primary Assembly

TCTGCTGTAGAACCAATGTCCCATACAGGACCCCACGTGCCACAGGAACCAAAAAGTCACACGCAGCGAA**−**GA**C**GAAGACACAGGAGACAACCTGTGTGGACAGCACAGAGCCACCTGCCCAGGACACCAATGGAGCCACAGGTGCAATTC


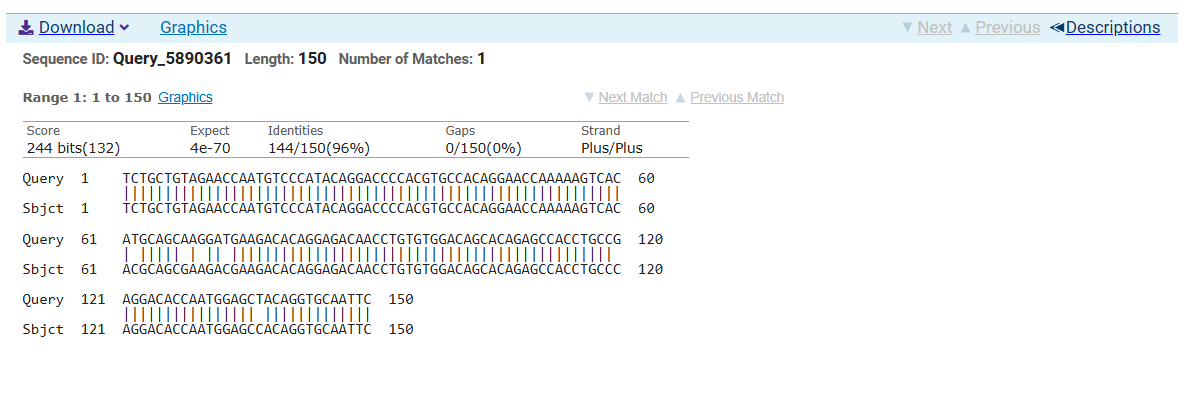


2 mismatches in SBE-primer

Detected allele: T

4) Chr15: 20523936-20524085

>ref|NC_000015.10|:20523936-20524085 Homo sapiens chromosome 15, GRCh38.p14 Primary Assembly

GAATTGCACCTGTGGCTCCATTGGTGTCCTGGGCAGGTGGCTCTGTGCTGTCCACACAGGTTGTCTCCTGTGTCTTC**G**TC**T**TCGCTGCGTGTGACTTTTTGGTTCCTGTGGCACGTGGGGTCCTGTATGGGACATTGGTTCTACAGCAGA


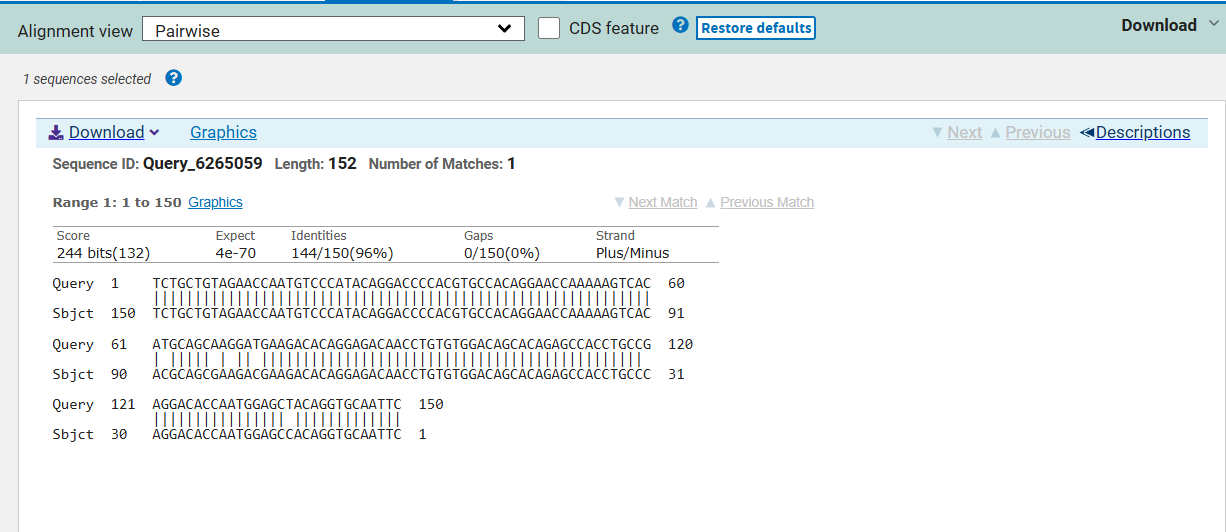


2 mismatches in SBE-primer

Detected allele: G

5) Chr15:23316610-23316759

>ref|NC_000015.10|:23316610-23316759 Homo sapiens chromosome 15, GRCh38.p14 Primary Assembly

GAATTGCACCTGTGGCTCCATTGGTGTCCTGGGCAGGTGGCTCTGTGCTGTCCACACAGGTTGTCTCCTGTGTCTTC**G**TC**T**TCGCTGCGTGTGACTTTTTGGTTCCTGTGGCACGTGGGGTCCTGTATGGGACATTGGTTCTACAGCAGA


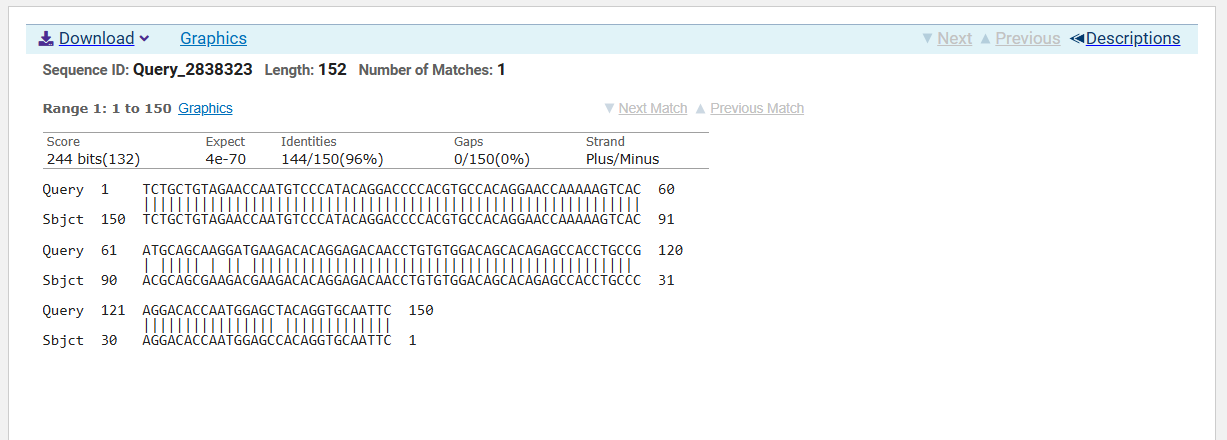


2 mismatches in SBE-primer

Detected allele: G

6) Chr15:28339927-28340076

>ref|NC_000015.10|:28339927-28340076 Homo sapiens chromosome 15, GRCh38.p14 Primary Assembly

GAATTGCACCTGTGGCTCCATTGGTGTCCTGGGCAGGTGGCTCTGTGCTGTCCACACAGGTTGTCTCCTGTGTCTTC**G**TC**T**TCGCTGCGTGTGACTTTTTGGTTCCTGTGGCACGTGGGGTCCTGTATGGGACATTGGTTCTACAGCAGA


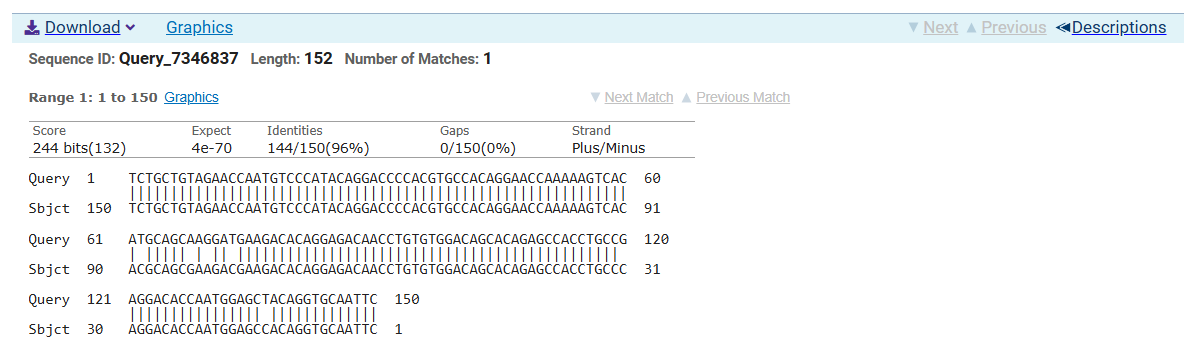


2 mismatches in SBE-primer

Detected allele: G

**Set 08: rs1667394**

Target PCR product

>ref|NC_000015.10|:28285002-28285131 Homo sapiens chromosome 15, GRCh38.p14 Primary Assembly

CACCATTAAGACGCAGCAATTCAAAACGTGCATACACCAAACAAACAAAGAAACAATGAATAGAACTGAAAGGAGAAAAAGACATTTATACTTTTATAGTTGGAGACCTCAAAGTCTCTCTCTACAGCTG

„High-risk“ off-target PCR products

1) Chr15:20469187-20469316

>ref|NC_000015.10|:20469187-20469316 Homo sapiens chromosome 15, GRCh38.p14 Primary Assembly

CACCATTAAGAC**A**CAGCAATTC**G**AAACGTGCA**G**ACACCAAACAAACAAAAAAACAATGAATAGAACCGAAAGGAGAAAAAGACATTTATACTTTTATAGTTGGAGACCTCAAAATCTCTCTCTACAGCTG


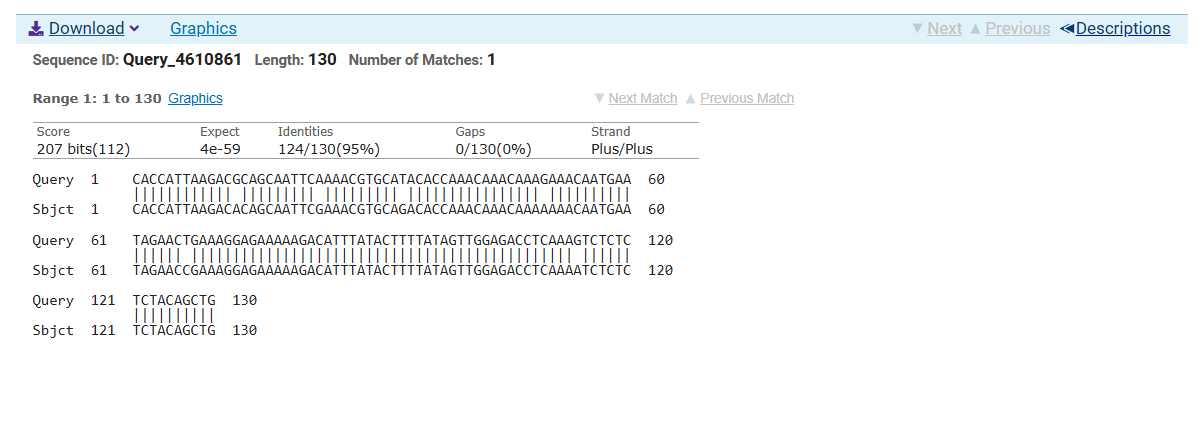


3 mismatches in SBE-primer

Detected allele: C

2) Chr15:22531122-22531251

>ref|NC_000015.10|:22531122-22531251 Homo sapiens chromosome 15, GRCh38.p14 Primary Assembly

CAGCTGTAGAGAGAGATTTTGAGGTCTCCAACTATAAAAGTATAAATGTCTTTTTCTCCTTTCGGTTCTATTCATTGTTTTTTTGTTTGTTTGGTGT**C**TGCACGTTT**C**GAATTGCTG**T**GTCTTAATGGTG


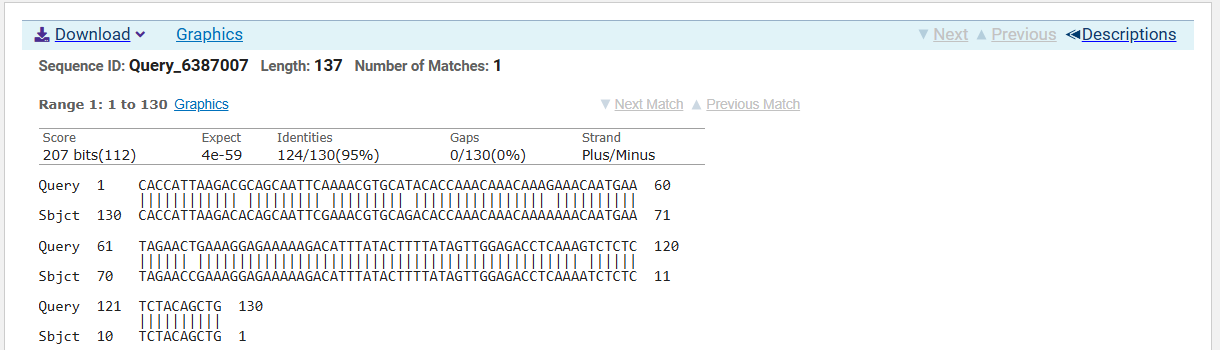


3 mismatches in SBE-primer

Detected allele: C

3) Chr15:28626332-28626461

>ref|NC_000015.10|:28626332-28626461 Homo sapiens chromosome 15, GRCh38.p14 Primary Assembly

CAGCTGTAGAGAGAGATTTTGAGGTCTCCAACTATAAAAGTATAAATGTCTTTTTCTCCTTTCGGTTCTGTTCATTGTTTTTTTGTTTGTTTGGTGT**C**TGCACGTTT**C**GAATTGCTG**T**GTCTTAATGGTG


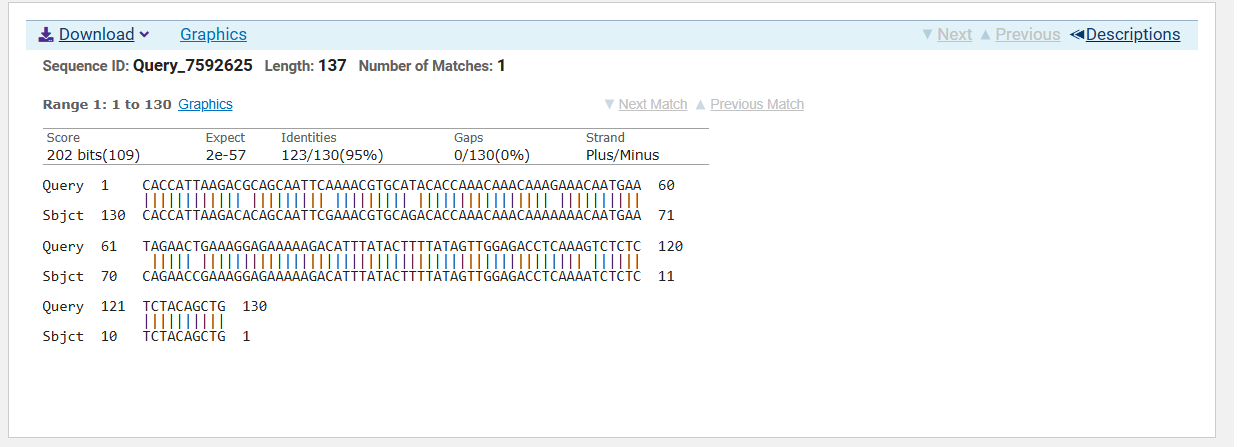


3 mismatches in SBE-primer

Detected allele: C

**Set 09: rs1126809**

Target PCR product

>ref|NC_000011.10|:89284739-89284838 Homo sapiens chromosome 11, GRCh38.p14 Primary Assembly

TGTTTCTTAGTCTGAATAACCTTTTCCTCTGCAGTATTTTTGAGCAGTGGCTCCGAAGGCACCGTCCTCTTCAAGAAGTTTATCCAGAAGCCAATGCACC

„High-risk“ off-target PCR products

1) Chr11:49415689-49415788 (TYRL, pseudogene)

>ref|NC_000011.10|:49415689-49415793 Homo sapiens chromosome 11, GRCh38.p14 Primary Assembly

GGTGCATTGGCTTCCGGATAAACTTCTTGAAGAAGACGGTGCCTTCGGAGCCACTGCTCAAAAATACTGCAGAGGAAAGGGTTATTCAGACTAAGAAACA


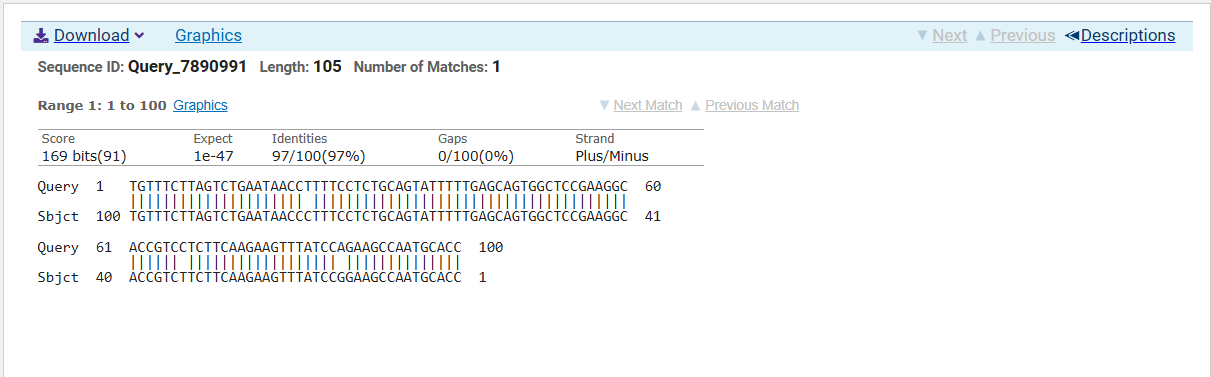


0 mismatches in SBE-primer

Detected allele: G
